# Efficacy and safety of donanemab in the European eligible population: TRAILBLAZER-ALZ 2 post-hoc analyses

**DOI:** 10.1016/j.tjpad.2026.100605

**Published:** 2026-05-27

**Authors:** Frank Jessen, Grazia Dell’Agnello, Jennifer A. Zimmer, Christophe Sapin, Sascha Dichter, Erin Doty, Stéphane Epelbaum, Cynthia D. Evans, Paula M. Hauck, Rashna Khanna, Dawn A. Brooks, John R. Sims, Federica Agosta

**Affiliations:** a Department of Psychiatry, Medical Faculty, University of Cologne, Cologne, Germany; b German Center for Neurodegenerative Diseases, Bonn, Germany; c Excellence Cluster on Cellular Stress Responses in Aging-Associated Diseases, University of Cologne, Cologne, Germany; d Eli Lilly and Company, Indianapolis, IN, USA; e Unit of Neurology and Neuroimaging of Neurodegenerative Diseases, IRCCS Ospedale San Raffaele, Milano, Italy; f “Vita-Salute San Raffaele” University, Milano, Italy

**Keywords:** Apolipoprotein E, Alzheimer’s disease, Donanemab, EU-eligible population

## Abstract

**Background:**

In the European Union (EU), donanemab is indicated in adults with early symptomatic Alzheimer’s disease who are apolipoprotein E ε4 non-carriers or heterozygotes. Among these, patients without superficial siderosis at baseline, uncontrolled hypertension, or anticoagulant use are eligible.

**Objective:**

To assess efficacy and safety of donanemab in the EU-eligible population.

**Methods:**

A post-hoc conservative hybrid imputation method was implemented for clinical efficacy analyses during the TRAILBLAZER-ALZ 2 placebo-controlled period. In the 78-week long-term extension (LTE) participants in the early-start (randomised to donanemab) and delayed-start (randomised to placebo with donanemab initiation during the LTE) groups were compared to a propensity-weighted external control. Participants were switched to placebo after meeting amyloid-based treatment course completion criteria.

**Results:**

By 76 weeks, donanemab-treated participants in the EU-eligible population had a mean Clinical Dementia Rating Scale (CDR)-Sum of Boxes change from baseline difference from placebo of -0.7 points (95% confidence interval, -1.0, -0.4) and a 40.3% lower risk of disease progression to the next stage (per CDR-Global score). Treatment benefit increased over 154 weeks for non-carriers and heterozygotes, including those meeting treatment course completion criteria by 52 or 76 weeks. In the placebo-controlled period, 119 (19.5%) and 49 (8.0%) donanemab-treated eligible participants experienced amyloid-related imaging abnormalities-edema/effusion and infusion-related reactions, respectively. Safety findings were similar among donanemab-treated participants in the placebo-controlled period and LTE delayed-start group.

**Conclusions:**

Consistent with previous TRAILBLAZER-ALZ 2 and LTE findings, donanemab significantly slowed disease progression compared to controls with a manageable safety profile in non-carriers and heterozygotes.

## Highlights

• Donanemab significantly slowed AD progression in the EU-eligible population.
• Treatment benefit with donanemab increased over 3 years.
• Clinical benefits continued to increase after treatment course completion.
• 52-week treatment course completers maintained amyloid clearance over 2 years.
• Lower ARIA rates with exclusion of *APOE* ɛ4 homozygotes (TRAILBLAZER-ALZ 2 posology).

## 1 Introduction

After decades of research in Alzheimer’s disease (AD), disease-modifying treatments are now available. Donanemab, a monoclonal antibody directed against a form of β-amyloid that is only present in brain amyloid plaques (the accumulation of which is a hallmark pathology of AD), has demonstrated significant slowing of cognitive and functional decline in people with early symptomatic AD (AD with mild cognitive impairment or mild dementia) [1,2].

Donanemab has received marketing authorisation in several countries and regions including the United States, Japan, China, the United Kingdom (UK) [3], and the European Union (EU) [4,5]. Although the EU- and UK-indicated populations are similar in that donanemab treatment is approved only for non-carriers or heterozygotes of the apolipoprotein E (*APOE*) ε4 allele, eligibility in clinical practice differs slightly due to modest differences in contraindications. In the EU, these include superficial siderosis, concomitant use of anticoagulants, and uncontrolled hypertension.

The rationale for the exclusion of patients who are homozygous for the *APOE* ε4 allele is that the risk of amyloid-related imaging abnormalities (ARIA), a known treatment-related adverse event (AE) with this class of amyloid-targeting therapies, increases with increasing number of *APOE* ε4 alleles [2,6].

The main objective of this post-hoc analysis was to analyse the treatment effects of donanemab in the EU-eligible population during the placebo-controlled period of the TRAILBLAZER-ALZ 2 trial. Efficacy outcomes were assessed using the conservative statistical approach for handling missing data required by the Committee for Medicinal Products for Human Use of the European Medicines Agency (EMA) for the EU-indicated population. Outcomes in the EU-indicated population are reported in the Supplement to allow evaluation of consistency with the EU-eligible population. Furthermore, analyses of the non-carrier and heterozygote population from the TRAILBLAZER-ALZ 2 long-term extension (LTE) were also conducted to provide long-term efficacy and safety data for donanemab in this population and are likewise presented in the Supplement.

This report complements the EU summary of product characteristics [5] by including additional efficacy and safety data, such as data on the EU-eligible population, which can enhance benefit–risk and treatment decision-making discussions between clinicians and their patients.

## 2 Methods

### 2.1 Participants and study design

The phase 3 TRAILBLAZER-ALZ 2 trial (NCT04437511) was a randomised, double-blind, multicentre, placebo-controlled study that assessed the efficacy and safety of donanemab for the treatment of early symptomatic AD (mild cognitive impairment or mild dementia due to AD) over 76 weeks [2]. Treatment course completion criteria (with success resulting in a blinded switch to placebo) and additional details, such as trial eligibility criteria and dosing information, can be found in the primary report [2].

The EU-indicated population included *APOE* ε4 non-carriers or heterozygotes. The EU-eligible population excluded those of the indicated population with superficial siderosis, anticoagulant use, or uncontrolled hypertension (for the purposes of these analyses, systolic and diastolic blood pressure while seated of ≥140 and ≥90 mm Hg, respectively) at baseline.

Participants who completed the placebo-controlled period of TRAILBLAZER-ALZ 2 were eligible to enrol in the LTE, a 78-week blinded study to assess the long-term efficacy and safety of donanemab. Details regarding the study design can be found in the Supplemental Methods and Zimmer et al. [7].

### 2.2 Efficacy analyses

Clinical outcome assessments, including the integrated AD Rating Scale (iADRS; range 0–144, lower scores indicate greater impairment), Clinical Dementia Rating Scale–Sum of Boxes (CDR-SB; range 0–18, higher scores indicate greater impairment), 13-item cognitive subscale of the Alzheimer’s Disease Assessment Scale (ADAS-Cog_13_; range 0–85, higher scores indicate greater impairment), Alzheimer’s Disease Cooperative Study–Instrumental Activities of Daily Living (ADCS-iADL; range 0–59, lower scores indicate greater impairment), and the Mini–Mental State Examination (MMSE; range 0–30, lower scores indicate greater impairment), were conducted in both the EU-eligible and EU-indicated subsets of the randomised TRAILBLAZER-ALZ 2 population [2]. Participants with low-medium tau pathology (as determined by 18F-flortaucipir positron emission tomography; see Sims et al. [2] were also assessed.

A mixed model for repeated measures was used to assess all clinical outcomes in the EU-indicated and EU-eligible populations, as well as the low-medium tau-indicated subpopulation. This model included treatment, visit, baseline tau category, pooled investigator, and concomitant use of symptomatic treatment (acetylcholinesterase inhibitors and/or memantine) as factors; baseline age and baseline score as covariates; and treatment-by-visit and baseline score-by-visit interaction terms.

For change from baseline differences from placebo in clinical scales, the EMA requested implementation of a hybrid imputation method for handling missing values that combines both jump-to-reference (J2R) and copy increment reference (CIR) imputation methods [8,9]. The J2R method replaces a missing value with the reference (i.e., placebo cohort) value at that timepoint. The CIR method replaces a missing value with a value that has been estimated to reflect observed trends (changes over time) in the reference arm. As the imputed values are derived from the reference cohort, both methods minimise the differences due to treatment effect and thus are considered more conservative compared to the original methodology (which does not employ any imputation). The J2R method was used for missing values due to serious, severe, or symptomatic ARIA (ARIA includes ARIA–edema/effusion [ARIA-E] and ARIA–microhemorrhages and hemosiderin deposits [ARIA-H]), or deaths, while the CIR method was applied for missing values due to all other causes.

As there was no internal placebo control in the LTE, participants from the Alzheimer’s Disease Neuroimaging Initiative (ADNI) were utilised as an external comparison cohort to evaluate efficacy (see also Zimmer et al. [7] and Supplemental Methods). A propensity score weighting method was applied to ensure that baseline disease characteristics of the TRAILBLAZER-ALZ 2 arms were balanced with the external control population from the ADNI used for comparison. A full description of the propensity score weighting method was previously published [7]. Selection of an EU-eligible propensity-weighted ADNI control group was not possible because superficial siderosis, anticoagulant use, and blood pressure were not collected as part of the ADNI data set. Therefore, assessments of an EU-eligible population during the LTE period were not conducted. However, results from both the EU-indicated population and the LTE population can be found in the Supplement for reference. In addition, the simultaneous application of both propensity score reweighting and multiple imputation with J2R /CIR is not methodologically possible. Therefore, the hybrid imputation methodology was not applied to any LTE analyses.

The CDR–Global (CDR-G) score was used to analyse the risk of progressing to the next stage of disease or to moderate AD dementia (CDR-G score ≥2). CDR-G total scores range from 0 (no dementia) to 3 (severe dementia), with higher scores indicating greater impairment. A Cox proportional hazards model was used to estimate the hazard ratio (HR) of progression to the next clinical stage (defined as any increase in the CDR-G score at two consecutive visits from baseline) and to moderate or severe dementia (defined as a CDR-G score of ≥2 at two consecutive visits from baseline for participants with a baseline CDR-G score of ≤1). Finally, the time-progression model for repeated measures was used to estimate the difference in time taken for participants in the placebo arm to reach the same change in score that was observed in the donanemab arm at the conclusion of the placebo-controlled period of the trial, as described in Sims et al. [2].

The adjusted means, standard errors (SEs), 95% confidence intervals (CIs), and *p* values for change from baseline in Centiloids (CL) were estimated using amyloid positron emission tomography and a mixed model for repeated measures with treatment, visit, and treatment-by-visit interaction as fixed factors, and score, score-by-visit interaction, age, and tau category as baseline covariates. Amyloid clearance was defined as achieving an amyloid plaque level <24.1 CL.

All analyses were post-hoc in nature and were not controlled for multiplicity. Most statistical analyses were done with SAS version 9.4. Some time-based progression analyses were analysed with R version 4.3.0.

### 2.3 Safety analyses

Safety was evaluated in all participants exposed to the study drug. All analyses were descriptive.

## 3 Results

### 3.1 TRAILBLAZER-ALZ 2: 76-week placebo-controlled treatment period

#### 3.1.1 Participants

Of the 1736 participants randomised in the 76-week, placebo-controlled treatment period of the TRAILBLAZER-ALZ 2 study, 1447 participants were *APOE* ε4 non-homozygotes. Demographics and baseline characteristics, including the baseline iADRS score, were generally similar between the placebo and donanemab treatment arms in the EU-eligible population (Table 1). A results section pertaining to the EU-indicated population and the LTE period of TRAILBLAZER-ALZ 2 can be found in the Supplement. Notably, 80% of participants in the EU-indicated and EU-eligible populations fell within a 36-point range on the iADRS (range, 85–121), covering about one-quarter of the total score range (0–144).

**Table 1:** Demographics and baseline characteristics of the EU-eligible population.

	EU-eligible population	
Variable	Placebo (*N* = 604)	Donanemab (*N* = 614)
Sex, n (%)		
Female	358 (59.3)	376 (61.2)
Male	246 (40.7)	238 (38.8)
Age, mean (SD), years	73.5 (6.1)	73.3 (6.3)
Country, n (%)		
Australia	2 (0.3)	10 (1.6)
Canada	55 (9.1)	51 (8.3)
Czech Republic	10 (1.7)	5 (0.8)
Japan	28 (4.6)	35 (5.7)
Netherlands	3 (0.5)	6 (1.0)
Poland	62 (10.3)	57 (9.3)
United Kingdom	15 (2.5)	10 (1.6)
United States	429 (71.0)	440 (71.7)
Race, n (%)^a^		
Asian	31 (5.1)	45 (7.3)
Black or African American	13 (2.2)	14 (2.3)
White	560 (92.7)	553 (90.2)
American Indian or Alaska Native	0	1 (0.2)
Missing	0	1 (0.2)
Ethnicity, n (%)^b^		
Hispanic/Latino	26 (6.1)	27 (6.2)
Not Hispanic/Latino	403 (93.9)	411 (93.8)
Education of ≥13 years, n (%)	435 (72.1)	426 (69.4)
*APOE* ε4 carrier, n (%)	394 (65.6)	399 (65.2)
ε2/ε2	1 (0.2)	0
ε2/ε3	18 (3.0)	16 (2.6)
ε2/ε4	22 (3.7)	18 (2.9)
ε3/ε3	188 (31.3)	197 (32.2)
ε3/ε4	372 (61.9)	381 (62.3)
ε4/ε4	—	—
Missing	3 (0.5)	2 (0.3)
AChEI/memantine use, n (%)	366 (60.6)	369 (60.1)
**Clinical scales^c^**		
iADRS score, mean (SD)	103.5 (14. 5)	104.7 (14.4)
CDR-SB score, mean (SD)	4.0 (2.1)	3.9 (2.1)
ADAS-Cog_13_ score, mean (SD)	29.3 (9.2)	28.3 (9.0)
ADCS-ADL total score, mean (SD)	66.3 (8.5)	66.5 (8.5)
ADCS-iADL score, mean (SD)	47.8 (8.0)	48.0 (7.9)
MMSE total score, mean (SD)^d^	22.1 (4.0)	22.4 (3.9)
MMSE category, n (%)^e^		
≥27	98 (16.3)	97 (15.8)
20–26	505 (83.7)	516 (84.0)
<20	0	1 (0.2)
CDR-G score, n (%)		
0	2 (0.3)	2 (0.3)
0.5	367 (61.2)	366 (60.4)
1	215 (35.8)	219 (36.1)
2	16 (2.7)	19 (3.1)
**Biomarker measures, mean (SD)**		
Amyloid plaque level, Centiloids^e,f^	101.6 (33.6)	102.6 (34.2)
Tau^e,g^	1.35 (0.27)	1.34 (0.26)

Notes: The EU-eligible population excluded participants with superficial siderosis, anticoagulant use, and uncontrolled BP (systolic BP≥140 mmHg and diastolic BP≥90 mmHg). Numbers of participants with non-missing data were used as denominators to calculate percentages. This analysis included participants with missing *APOE* ε4 genotype data.

a Participants self-reported race data within fixed categories. ^b^Ethnicity reporting was limited to only participants in the United States/Puerto Rico. ^c^Clinical outcome ranges were as follows: ADAS-Cog_13_ scores range from 0 to 85, with higher scores indicating greater overall cognition deficit; ADCS-ADL scores range from 0 to 78, with lower scores indicating greater level of impairment; ADCS-iADL scores range from 0 to 59, with lower scores indicating greater impairment in daily function; CDR-G scores range from 0 (no dementia) to 3 (severe dementia); CDR-SB scores range from 0 to 18, with higher scores indicating greater clinical impairment; iADRS scores range from 0 to 144, with lower scores indicating greater impairment; and MMSE scores range from 0 to 30, with lower scores indicating greater level of impairment. ^d^Last non-missing MMSE score before or at the start of study treatment. ^e^Based on screening data. ^f^Assessed with 18F-florbetapir or 18F-florbetaben PET scan. ^g^Assessed with 18F-flortaucipir PET scan. Global tau uptake was measured using a composite AD-signature weighted neocortical standardised uptake value ratio with white matter signal reference. AChEI: acetylcholinesterase inhibitor; AD: Alzheimer’s disease; ADAS-Cog_13_: 13-item cognitive subscale of the Alzheimer’s Disease Assessment Scale; ADCS-ADL: Alzheimer’s Disease Cooperative Study–Activities of Daily Living; ADCS-iADL: Alzheimer’s Disease Cooperative Study–Instrumental Activities of Daily Living; *APOE*: apolipoprotein E; BP: blood pressure; CDR-G: Clinical Dementia Rating Scale–Global; CDR-SB: Clinical Dementia Rating Scale–Sum of Boxes; EU: European Union; iADRS: integrated Alzheimer’s Disease Rating Scale; MMSE: Mini–Mental State Examination; N: number of participants in the analysis population; n: number of participants within each specific category; PET: positron emission tomography; SD: standard deviation.

#### 3.1.2 Efficacy

##### 3.1.2.1 Clinical outcomes

**Fig. 1:**
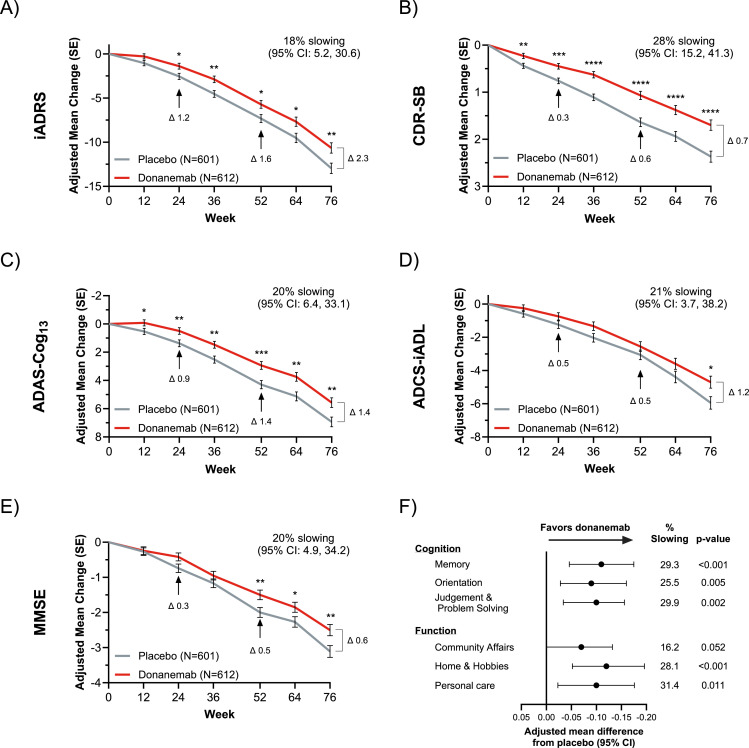
Clinical outcomes in the EU-eligible population.

At 76 weeks, the adjusted mean (SE) change from baseline in the iADRS score in the EU-eligible population using the hybrid imputation method was −13.0 (0.6) and −10.6 (0.6) in the placebo and donanemab arms, respectively (difference, 2.3 [95% CI: 0.7, 3.9]; *p*=0.005), representing an 18% (95% CI: 5.2, 30.6) slowing of disease progression (Fig. 1A). At 76 weeks, the adjusted mean change from baseline difference in the CDR-SB score between the donanemab and placebo arms was −0.7 (95% CI: −1.0, −0.4; *p*<0.001), with a 28% (95% CI: 15.2, 41.3) slowing of disease progression. (Fig. 1B). Similar results were observed with the ADAS-Cog_13_ (Fig. 1C), ADCS-iADL (Fig. 1D) and MMSE (Fig. 1E) clinical scales. A comparison of these results obtained using the hybrid imputation method to those obtained using the original statistical methodology is reported in Table 2.

Donanemab was significantly better than placebo in slowing disease progression at 76 weeks as measured with all five clinical scales: (A) iADRS; (B) CDR-SB; (C) ADAS-Cog_13_; (D) ADCS-iADL; and (E) MMSE. *p<0.05, **p<0.01, ***p<0.001, ****p<0.0001. (F) Assessment of individual CDR-SB domains at week 76. Notes: The EU-eligible population excluded participants with superficial siderosis, anticoagulant use, and uncontrolled BP (systolic BP ≥140 mmHg and diastolic BP ≥90 mmHg). Analyses for all panels except for (F) did not include participants with missing *APOE* ε4 genotype data. Analyses presented in all panels were conducted using the conservative methodology for handling missing data (multiple imputation with jump-to-reference and copy increment reference methods). ADAS-Cog_13_: 13-item cognitive subscale of the Alzheimer’s Disease Assessment Scale; ADCS-iADL: Alzheimer’s Disease Cooperative Study–Instrumental Activities of Daily Living; *APOE*: apolipoprotein E; BP: blood pressure; CDR-SB: Clinical Dementia Rating Scale–Sum of Boxes; CI: confidence interval; EU: European Union; iADRS: integrated Alzheimer’s Disease Rating Scale; MMSE: Mini–Mental State Examination; N: number of randomised participants; SE: standard error.

**Table 2:** Impact of imputation method on clinical outcomes in the EU-eligible population at week 76.

	Difference from placebo at 76 weeks in the EU-eligible population					
	Original statistical methodology^a^			Conservative hybrid imputation^b^		
Clinical outcome^c^	Adjusted mean difference (95% CI)	*p* value	Percent slowing^d^ (95% CI)	Adjusted mean difference (95% CI)	*p* value	Percent slowing^d^ (95% CI)
iADRS	2.52 (0.89, 4.14)^e^	0.002^e^	19.9% (7.0, 32.8)^e^	2.32 (0.69, 3.95)^f^	0.005^f^	17.9% (5.2, 30.6)^f^
CDR-SB	−0.73 (−1.03, −0.43)^f^	<0.001^f^	30.8% (17.7, 43.9)^f^	−0.67 (−0.97, −0.37)^f^	<0.001^f^	28.2% (15.2, 41.3)^f^
ADAS-Cog_13_	−1.64 (−2.53, −0.74)^e^	<0.001^e^	23.5% (10.5, 36.6)^e^	−1.37 (−2.28, −0.45)^f^	0.003^f^	19.7% (6.4, 33.1)^f^
ADCS-iADL	1.25 (0.24, 2.26)^e^	0.015^e^	21.8% (4.1, 39.5)^e^	1.24 (0.23, 2.26)^f^	0.016^f^	20.9% (3.7, 38.2)^f^
MMSE	0.73 (0.30, 1.16)^e^	<0.001^e^	23.9% (9.5, 38.3)^e^	0.61 (0.16, 1.06)^f^	0.009^f^	19.5% (4.9, 34.2)^f^

Notes: The EU-eligible population excluded participants with superficial siderosis, anticoagulant use, and uncontrolled BP (systolic BP≥140 mmHg and diastolic BP≥90 mmHg).

a No formal imputation was used. Analyses did not include participants with missing *APOE* ε4 genotype data. ^b^Conservative hybrid imputation was used: If the participant discontinued due to serious/severe/symptomatic ARIA-E/ARIA-H or death, the jump-to-reference method was used; the copy increment reference method was used for all other missing data. Analyses did not include participants with missing *APOE* ε4 genotype data. ^c^Clinical outcomes were scored as follows: ADAS-Cog_13_ scores range from 0 to 85, with higher scores indicating greater overall cognition deficit; ADCS-iADL scores range from 0 to 59, with lower scores indicating greater impairment in daily function; CDR-SB scores range from 0 to 18, with higher scores indicating greater clinical impairment; iADRS scores range from 0 to 144, with lower scores indicating greater impairment; and MMSE scores range from 0 to 30, with lower scores indicating greater level of impairment. ^d^Percent slowing was calculated by dividing the adjusted mean CFB treatment differences at 76 weeks by the adjusted mean CFB with placebo at 76 weeks and multiplying by 100. The 95% CIs were estimated using the Delta method. ^e^NCS2: Adjusted mean change from baseline, SE, 95% CI, and *p* values were derived using the NCS model with 2 degrees of freedom. The model was adjusted for basis expansion terms (two terms), basis expansion term-by-treatment interaction and covariates for age at baseline, pooled investigator, baseline tau category, and baseline AChEI/memantine use. ^f^MMRM: Adjusted mean CFB, 95% CIs, and *p* values were derived using an MMRM with treatment, visit, baseline tau category, pooled investigator, and concomitant use of symptomatic treatment (acetylcholinesterase inhibitors and/or memantine) as factors, baseline age and baseline score as covariates, and treatment-by-visit and baseline score-by-visit interaction terms. The 95% CIs for adjusted mean changes were calculated with the normal approximation method. AChEI: acetylcholinesterase inhibitor; ADAS-Cog_13_: 13-item cognitive subscale of the Alzheimer’s Disease Assessment Scale; ADCS-iADL: Alzheimer’s Disease Cooperative Study–Instrumental Activities of Daily Living; *APOE*: apolipoprotein E; ARIA-E: amyloid-related imaging abnormality–edema/effusion; ARIA-H: amyloid-related imaging abnormality–microhemorrhages and hemosiderin deposits; BP: blood pressure; CDR-SB: Clinical Dementia Rating Scale–Sum of Boxes; CFB: change from baseline; CI: confidence interval; EU: European Union; iADRS: integrated Alzheimer’s Disease Rating Scale; MMRM: mixed model for repeated measures; MMSE: Mini–Mental State Examination; NCS: natural cubic spline.

###### 3.1.2.1.1 CDR-SB domains

Donanemab was associated with overall better outcomes versus placebo on all individual CDR-SB domains with statistically significant separation on five out of six domains. For the cognitive domains, the percent slowing was 29.3% (*p*<0.001) for memory, 25.5% (*p*=0.005) for orientation, and 29.9% (*p*=0.002) for judgement and problem solving. The percent slowing for functional domains was 28.1% (*p*<0.001) for home and hobbies and 31.4% (*p*=0.011) for personal care (Fig. 1F). Although the treatment effect for community affairs showed a favourable trend for donanemab (16.2% slowing), the difference did not reach statistical significance (p=0.052) (Fig. 1F).

###### 3.1.2.1.2 Disease progression to the next clinical stage

The risk of disease progression to the next clinical stage of disease, as assessed by the CDR-G, was 40.3% lower with donanemab (*N*=581; 174 events) compared with placebo (*N*=581; 247 events) over the 76-week trial in the EU-eligible population (HR=0.597 [95% CI: 0.5, 0.8]; *p*<0.001) (Table 3). The risk of progression to moderate dementia (CDR-G score ≥2) was significantly reduced by 47.2% (donanemab, *N*=565 [41 events]; placebo, *N*=567 [65 events]; HR=0.528 [95% CI: 0.3, 0.9]; *p*=0.024).

**Table 3:** Additional clinical and biomarker outcomes in the EU-eligible population.

	EU-eligible population	
Category	Placebo	Donanemab
**Clinical outcomes**		
**Hazard ratio for progression on CDR-G**^a^		
CDR-G score (95% CI)^b,c^	—	0.597 (0.473, 0.754)
*p* value vs placebo		<0.001
Number of events/n	247/581	174/581
CDR-G score ≥2 (95% CI)^d^	—	0.528 (0.303, 0.919)
*p* value vs placebo		0.024
Number of events/n	65/567	41/565
**No progression as measured by CDR-SB^d,e^**		
**at 52 weeks**		
n/N	117/478	193/481
Estimated percent of no progression (95% CI)	22% (0.19, 0.26)	37% (0.3, 0.4)
*p* value vs placebo	—	<0.001
**at 76 weeks**		
n/N	91/451	128/439
Estimated percent of no progression (95% CI)	18% (0.15, 0.22)	25% (0.2, 0.3)
*p* value vs placebo	—	0.006
**Biomarker outcomes at 76 weeks**^d^		
**Amyloid (CL)**^f^		
n	601	612
Adjusted mean CFB difference vs placebo	—	−75.8
95% CI	—	(−79.4, −72.2)
*p* value	—	<0.001
**Amyloid <24.1 CL at 76 weeks**^d^		
n/N	0/464	363/451
Percentage (95% CI)^g^	0.0% (0.0, 0.8)	80.5% (76.6, 83.9)
*p* value^h^		<0.001

Notes: The EU-eligible population excluded participants with superficial siderosis, anticoagulant use, and uncontrolled BP (systolic BP≥140 mmHg and diastolic BP≥90 mmHg).

a CDR-G scores range from 0 to 3 as follows: 0 = no impairment, 0.5 = mild cognitive impairment, 1 = mild dementia, 2 = moderate dementia, and 3 = severe dementia; ≥2 means progression to moderate or severe dementia. Hazard ratio, 95% CI, and *p* value were calculated using a Cox proportional hazards model stratified by pooled investigator and baseline tau level that included baseline covariates of age, AChEI/memantine use, and clinical outcome score. ^b^Discontinuations due to death and ARIA are counted as events. ^c^Analysis included participants with missing *APOE* ε4 genotype data. ^d^Analysis did not include participants with missing *APOE* ε4 genotype data. ^e^CDR-SB scores range from 0 to 18, with higher scores indicating greater clinical impairment. No progression was defined as a CDR-SB score CFB of ≤0. Probability of no progression, 95% CI, and *p* value were derived using a generalized mixed model with factors for treatment, visit, and treatment-by-visit interaction and baseline covariates of age, tau category, AChEI/memantine use, clinical outcome score, and clinical outcome score-by-visit interaction. ^f^Hybrid imputation method implemented (if the participant discontinued due to serious/severe/symptomatic ARIA-E/ARIA-H or death, the jump-to-reference method was used; the copy increment reference method was used for all other missing data). Adjusted mean change from baseline, 95% CI, and *p* value were derived using a mixed model for repeated measures with fixed factors for treatment, visit, and treatment-by-visit interaction and baseline covariates of age, tau category, clinical outcome score, and clinical outcome score-by-visit interaction. ^g^95% CIs were calculated using the Wilson score method. ^h^*p* value from one sample frequency test evaluating if the percent amyloid negative equals 0. AChEI: acetylcholinesterase inhibitor; *APOE*: apolipoprotein E; BP: blood pressure; CDR-G: Clinical Dementia Rating Scale–Global; CDR-SB: Clinical Dementia Rating Scale–Sum of Boxes; CFB: change from baseline; CI: confidence interval; CL: Centiloids; EU: European Union; N: number of participants in the analysis population; n: number of participants within each specific category.

###### 3.1.2.1.3 Disease stabilisation

An estimated 37% of donanemab-treated participants in the EU-eligible population were shown to be clinically stable (showed no decline on the CDR-SB from baseline) at 52 weeks compared with 22% of participants who received placebo (*p*<0.001). Furthermore, 25% of donanemab-treated participants versus 18% of participants who received placebo had not progressed by 76 weeks (*p*=0.006) (Table 3).

###### 3.1.2.1.4 Time saved

**Fig. 2:**
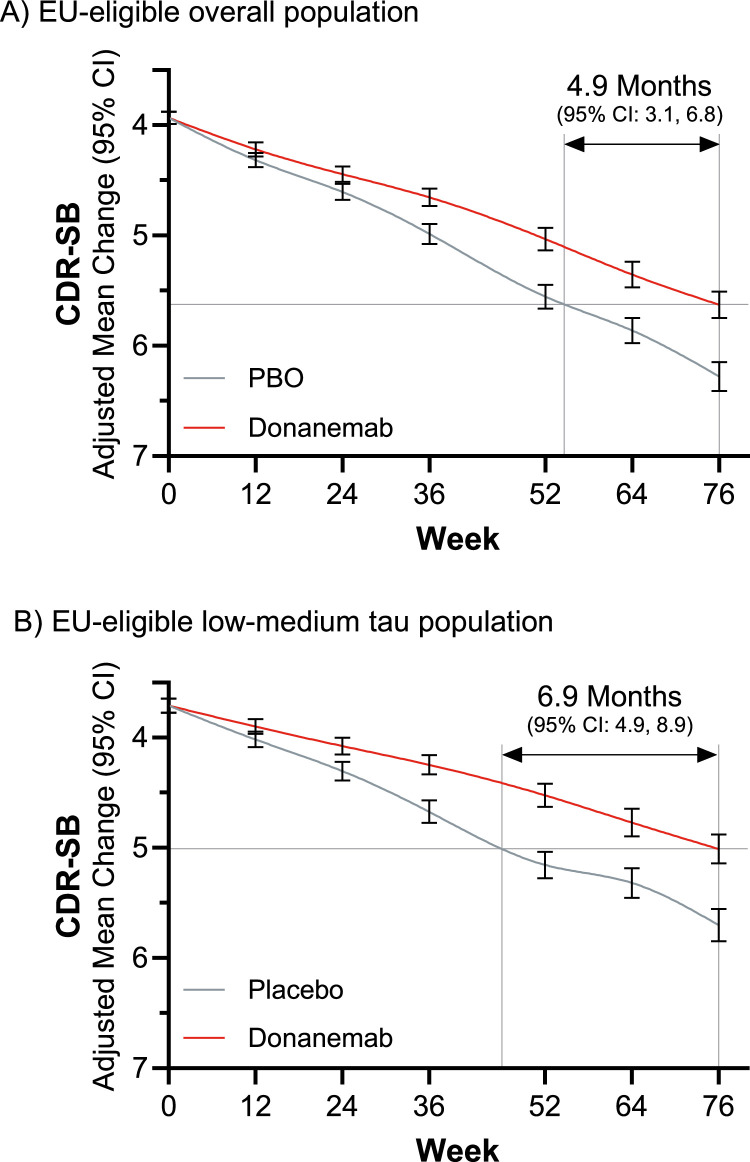
Time saved in the EU-eligible population.

At 76 weeks, disease progression among participants treated with donanemab in the EU-eligible population was delayed by 4.9 months (95% CI: 3.1, 6.8), as assessed by the CDR-SB (Fig. 2). Disease progression for the low-medium population was delayed by an additional 2 months (for a total of 6.9 months [95% CI: 4.9, 8.9]).

Results of the CDR-SB progression model for repeated measures analysis with the conservative hybrid imputation methodology, illustrating the time saved with donanemab treatment in (A) the EU-eligible overall population and (B) the EU-eligible low-medium tau population. Notes: The EU-eligible population excluded participants with superficial siderosis, anticoagulant use, and uncontrolled BP (systolic BP≥140 mmHg and diastolic BP≥90 mmHg). These analyses did not include participants with missing *APOE* ε4 genotype data. *APOE*: apolipoprotein E; CDR-SB: Clinical Dementia Rating Scale–Sum of Boxes; CI: confidence interval; EU: European Union; N: number of participants; n: number of participants at risk; SE: standard error.

##### 3.1.2.2 Biomarker outcomes

In the EU-eligible population, treatment with donanemab was significantly more effective than placebo in reducing amyloid plaque levels (adjusted mean change difference from placebo: −75.8 CL [95% CI: −79.4, −72.2]; *p*<0.001), resulting in 33.5%, 69.7%, and 80.5% of donanemab-treated participants achieving amyloid clearance (<24.1 CL) at weeks 24, 52, and 76, respectively (Table 3). Donanemab treatment significantly reduced plasma P-tau217 at all timepoints in the EU-eligible population (all *p*<0.001), with an adjusted mean change from baseline to 76 weeks (log_10_-based) difference from that of placebo of −0.20 (95% CI, −0.22, −0.17).

#### 3.1.3 Safety

In the EU-eligible population, treatment-emergent AEs (TEAEs) were reported by 79.8% and 87.5% of participants who received placebo and donanemab, respectively. The incidence of death with placebo and donanemab was 1.2% and 1.3%, respectively. There were no ARIA-related deaths reported in the EU-eligible population. Treatment discontinuation due to AEs was reported for 22 participants (3.6%) in the placebo arm and 73 participants (12.0%) in the donanemab arm. The most common TEAEs are shown in Table 4.

**Table 4:** Safety summary for the EU-eligible population.

	EU-eligible population	
Category, n (%)^a^	Placebo (*N* = 603)	Donanemab (*N* = 609)
**Overview^b^**		
Deaths^c^	7 (1.2)	8 (1.3)
Deaths associated with any ARIA/ICH >1 cm	0	0
Participants with ≥1 SAE	74 (12.3)	89 (14.6)
Treatment discontinuations due to AEs	22 (3.6)	73 (12.0)
Study discontinuations due to AEs	21 (3.5)	39 (6.4)
Participants with ≥1 TEAE	481 (79.8)	533 (87.5)
**TEAEs with ≥5% incidence in any arm^b,d^**		
ARIA-E	12 (2.0)	119 (19.5)
ARIA-H	39 (6.5)	100 (16.4)
COVID-19	106 (17.6)	95 (15.6)
Headache	66 (10.9)	89 (14.6)
Fall	68 (11.3)	85 (14.0)
Infusion-related reaction	1 (0.2)	49 (8.0)
Arthralgia	33 (5.5)	41 (6.7)
Diarrhoea	35 (5.8)	36 (5.9)
Superficial siderosis of central nervous system	4 (0.7)	34 (5.6)
Urinary tract infection	39 (6.5)	32 (5.3)
Dizziness	32 (5.3)	30 (4.9)
Fatigue	33 (5.5)	29 (4.8)
**ARIA overview**		
Any ARIA (ARIA-E or ARIA-H)^b,e^	75 (12.4)	195 (32.0)
SAE of any ARIA^f^	0	6 (1.0)
ARIA-E^b,e^	12 (2.0)	119 (19.5)
SAE of ARIA-E^b,f^	0	6 (1.0)
Asymptomatic^b^	12 (2.0)	89 (14.6)
Symptomatic^b^	0	30 (4.9)
Maximum radiographic severity^g,h^		
Mild	10 (1.7)	35 (5.8)
Moderate	1 (0.2)	76 (12.5)
Severe	0	6 (1.0)
ARIA-H^b.e^	67 (11.1)	161 (26.4)
SAE of ARIA-H^b,f^	0	0
Asymptomatic^b^	66 (10.9)	155 (25.5)
Symptomatic^b^	1 (0.2)	6 (1.0)
Maximum radiographic severity^g,h^		
Mild	51 (8.5)	81 (13.3)
Moderate	11 (1.8)	34 (5.6)
Severe	3 (0.5)	44 (7.2)
Isolated ARIA-H^b,e,i^	62 (10.3)	76 (12.5)
Microhemorrhage^b,g^	62 (10.3)	132 (21.7)
Superficial siderosis^b,g^	10 (1.7)	76 (12.5)
Macrohemorrhage^b,e^	1 (0.2)	2 (0.3)
SAE of macrohemorrhage^b,f^	0	0

Note: The EU-eligible population excluded participants with superficial siderosis, anticoagulant use, and uncontrolled BP (systolic BP≥140 mmHg and diastolic BP≥90 mmHg).

a Participants may have been counted in more than one category. ^b^Analyses included participants with missing *APOE* ε4 genotype data. **^c^**Deaths are also included as serious AEs and discontinuations due to AEs. ^d^TEAEs were coded using the Medical Dictionary for Adverse Events version 25.1. ^e^Based on MRI or TEAE cluster. ^f^Based on TEAE cluster. ^g^Based on MRI only. ^h^Analysis did not include participants with missing *APOE* ε4 genotype data ^i^Isolated ARIA-H means no ARIA-E events based on MRI or TEAE cluster in the same analysis period. AE: adverse event; *APOE*: apolipoprotein E; ARIA: amyloid-related imaging abnormalities; ARIA-E: amyloid-related imaging abnormality–edema/effusion; ARIA-H: amyloid-related imaging abnormality–microhemorrhages and hemosiderin deposits; BP: blood pressure; COVID-19: coronavirus disease 2019; EU: European Union; ICH: intracerebral hemorrhage; MRI: magnetic resonance imaging; N: number of participants in the analysis population; n: number of participants within each specific category; SAE: serious adverse event; TEAE: treatment-emergent adverse event.

The incidence of ARIA-E based on magnetic resonance imaging or TEAE cluster was 2.0% (12/603) and 19.5% (119/609) in the placebo and donanemab arms, respectively. The incidence of symptomatic and serious ARIA-E in donanemab-treated participants was 4.9% (30/609) and 1.0% (6/609), respectively. No cases of serious or symptomatic ARIA-E were reported in the placebo arm.

ARIA-H incidence was 11.1% (67/603) and 26.4% (161/609) in the placebo and donanemab arms, respectively, of which 0.2% (1/603) and 1.0% (6/609) were symptomatic, and none were serious in either arm. The proportion of participants with isolated ARIA-H was 10.3% and 12.5% in the placebo and donanemab arms, respectively. Infusion-related reactions were reported for one participant (0.2%) who received placebo and 49 (8.0%) who received donanemab (Table 4).

## 4 Discussion

In the TRAILBLAZER-ALZ 2 study, treatment with donanemab resulted in significant and clinically relevant slowing of cognitive and functional decline in participants with early symptomatic AD [2]. Following EMA approval of donanemab, additional analyses were conducted in the EU-eligible population—participants who were *APOE* ε4 non-carriers or heterozygotes and without superficial siderosis, anticoagulant use, or uncontrolled hypertension at baseline—reflecting the patients expected to be treated in clinical practice in Europe.

Primary and secondary endpoints in this EU-eligible population were evaluated using the post-hoc hybrid conservative imputation (J2R/CIR) —the same method requested by the EMA for assessing efficacy in the EU-indicated population—and the original statistical method. Across both analytical approaches, donanemab consistently demonstrated clinical benefit. Specifically, using the conservative imputation method in the EU-eligible population, donanemab showed statistically significant benefits compared with placebo across multiple clinical efficacy measures, including the iADRS, CDR-SB, ADAS-Cog13, ADCS-iADL, and MMSE, with an 18% to 28% slowing of disease progression compared to placebo at week 76.

When assessing treatment effect versus placebo, it is important to note that some clinical scales have a wide scoring range because they are intended to capture the disease continuum (i.e., from no impairment to severe AD) [10]. For example, the iADRS score ranges from 0 to 144. However, only a small portion of the iADRS scale is relevant for the interpretation of treatment effects in patients with mild cognitive impairment or mild dementia due to AD. For most EU-eligible participants of TRAILBLAZER-ALZ 2, baseline scores were clustered within a relatively narrow segment of the iADRS scale (36 points), and this restricted range (rather than the full 0—144 iADRS range) should be considered when interpreting the treatment effect.

The clinical efficacy demonstrated with donanemab in the TRAILBLAZER-ALZ 2 study aligns with patients’, caregivers’, and clinicians’ desire to slow clinical progression, enabling patients to remain at earlier, more highly functional clinical stages of disease for longer [11,12]. Donanemab-treated participants in the EU-eligible population had a significantly lower risk of progressing to the next clinical disease stage and to moderate disease over the 76-week placebo-controlled study as determined by the CDR-G score, and a significant proportion of participants who received donanemab remained stable at 52 weeks with no progression of disease as measured on the CDR-SB. Maintaining a milder stage of disease has a clinically meaningful impact on practical aspects of daily living for patients with AD and their care partners, including the ability to maintain their current level of independence, spend more time with loved ones, and participate in activities that matter most to them for longer. Along with the individual-level benefits, remaining at a milder stage for additional time also translates into meaningful societal advantages. As noted by the European Alzheimer’s Disease Consortium, “from the societal point of view, this risk reduction is important given the increase of care-related costs with advancing disease stages” [13].

The benefit of donanemab cannot be appropriately characterised without integrating safety considerations. In the EU-eligible population, the overall frequency of ARIA events was reduced by approximately 5% compared with the overall TRAILBLAZER-ALZ 2 study population (32.0%vs 36.8%, respectively) [2].The enhanced safety in the EU-eligible population is also reflected by the majority of ARIA-E events experienced by participants treated with donanemab being asymptomatic (74.8%) and of mild to moderate radiographic severity (94.9%). In addition, nearly half of the observed ARIA-H events were isolated. Isolated ARIA-H events were reported for 10.3% and 12.5% of participants who received placebo and donanemab, respectively, reflecting the risk associated with the underlying disease even in the absence of donanemab treatment. No deaths related to ARIA and/or intracerebral hemorrhage occurred in the EU-eligible population.

Although the safety profile of donanemab was enhanced in the EU-eligible population, the clinical benefits were consistent with those observed in the broader overall population of TRAILBLAZER-ALZ 2 [2]. For example, donanemab-mediated slowing of disease progression, as measured by CDR-SB, was 28.2% and 30.8% in the EU-eligible population with and without the hybrid imputation method respectively, compared to 28.9% in the overall TRAILBLAZER-ALZ 2 population (without imputation). The reduced ARIA risk, together with significant efficacy, confirms a positive risk–benefit profile in the EU-eligible population. Notably, in the phase 3 TRAILBLAZER-ALZ 6 study evaluating different dosing regimens of donanemab in adults with early symptomatic AD, modified titration resulted in an even lower percentage of participants reporting ARIA-E, corresponding to a 41% relative risk reduction in the study population at 24 weeks [14,15]. This more gradual titration corresponds to the dosing regimen approved by the EMA [5].

The results of the LTE reported in the Supplement provide evidence supporting the favourable benefit–risk profile in *APOE* ɛ4 non-carriers and heterozygotes over the long term. An increasing treatment effect was demonstrated over 154 weeks for donanemab-treated participants compared to the untreated propensity-weighted ADNI cohort, including for early-start participants who met treatment completion criteria by 52 weeks and 76 weeks. These findings support the ability of donanemab to successfully modify the course of disease in *APOE* ɛ4 non-carriers and heterozygotes similarly to the overall study population and demonstrate the durability of the treatment effect of donanemab with limited-duration dosing.

Additional efficacy results from the LTE show that treating AD earlier on the disease continuum is more likely to result in better long-term outcomes. At 154 weeks, participants who were treated earlier with donanemab (early-start group) demonstrated a 29% lower risk of progressing to the next stage of disease than participants who started donanemab treatment later (delayed-start group), as assessed by the CDR-G score. Progression to the next stage of disease is more appropriate for comparing the early-start and delayed-start groups over the whole study period than directly comparing the mean changes from baseline. The requirement of two consecutive visits to characterize progression makes this outcome less sensitive to random fluctuations compared to mean change from baseline. The appropriate statistical model for such an outcome measure is a Cox proportional hazard model. Risk of progression to next stage of disease also supports ease of communication of treatment efficacy to people living with AD and their care partners, as any change in the CDR-G score reflects a clinically meaningful shift. The clinical efficacy of donanemab observed during the LTE period is supported by the robust amyloid reduction in both early- and delayed-start groups at 76 weeks after donanemab treatment was initiated and by the similar percentage of participants who achieved amyloid clearance at each time point. After completing donanemab treatment, the rate of amyloid reaccumulation was comparable to that seen with the natural history of the disease [16,17].

Importantly, the safety findings observed in the LTE were consistent with the previously established safety profile, with safety of the delayed-start group generally similar to that of the donanemab arm during the first 76 weeks of treatment.

Participants who did not meet treatment course completion criteria and thus continued to receive donanemab for more than 76 weeks, showed reduced frequencies of ARIA and infusion-related reactions during the LTE compared with donanemab-treated participants during the placebo-controlled period.

All analyses in this report are post-hoc, exploratory, and not controlled for multiplicity. Exclusion of *APOE* ɛ4 homozygotes and those with contraindications resulted in a smaller subpopulation than in the primary study [2]. Additionally, the LTE study was not designed to include an internal placebo comparator [7]. The external comparison cohort may have differed from the LTE cohort due to variations in study conduct and assessments, time periods and geographic regions of data collection, and other potential unmeasured confounding factors. Differences in disease severity between participants in the early- and delayed-start groups at the time of donanemab initiation makes comparisons between these two groups challenging due to potential bias. Furthermore, the rate of clinical decline [18,19] and treatment benefit varies depending on disease stage [2]. It is also important to note that the study design transitioned participants to placebo once treatment completion criteria were met. As a result, most (74.8%) of the early-start participants received placebo for the entirety of the LTE. The complexities of the study design, population differences, and unequal treatment exposure, should be considered when interpreting the LTE data. Lastly, all results included reflect the TRAILBLAZER-ALZ 2 donanemab dosing regimen, whereas a more gradual titration will be used in clinical practice.

## 5 Conclusions

Donanemab demonstrates a favourable benefit-risk profile in the EU-eligible population, with efficacy supported by robust clinical and biomarker outcomes using multiple endpoints and an enhanced safety profile. Most ARIA events were clinically asymptomatic and radiographically mild to moderate in severity compared with the overall population.

## Funding

This work was supported by Eli Lilly and Company.

## Authorship / credit authorship contribution statement

Writing -Review and editing was conducted by Frank Jessen, Grazia Dell’Agnello, Jennifer A. Zimmer, Christophe Sapin, Sascha Dichter, Erin Doty, Stéphane Epelbaum, Cynthia D. Evans, Paula M. Hauck, Rashna Khanna, Dawn A. Brooks, John R. Sims, and Federica Agosta.

Validation and formal analyses were conducted by Christophe Sapin.

The original draft was written by Grazia Dell’Agnello and Paula Hauck.

Visualizations were created by Paula Hauck.

## Declaration of the use of generative AI and AI-assisted technologies in scientific writing and in figures, images and artwork

No generative AI or AI-assisted technologies were used in the preparation of this manuscript.

## Ethical statement

The TRAILBLAZER-ALZ 2 phase 3 trial (ClinicalTrials.gov Identifier: NCT04437511) was conducted according to the Declaration of Helsinki, the International Conference on Harmonization Good Clinical Practice guidelines, and local regulatory requirements. An independent ethics committee/institutional review board at each site approved the study protocols. Participants and study partners provided written consent. No animal studies were conducted for this report.

## Data statement

Lilly provides access to all individual participant data collected during the trial, after anonymization, with the exception of pharmacokinetic or genetic data. Data are available to request 6 months after the indication studied has been approved in the US and EU and after primary publication acceptance, whichever is later. No expiration date of data requests is currently set once data are made available. Access is provided after a proposal has been approved by an independent review committee identified for this purpose and after receipt of a signed data sharing agreement. Data and documents, including the study protocol, statistical analysis plan, clinical study report, blank or annotated case report forms, will be provided in a secure data sharing environment. For details on submitting a request, see the instructions provided at www.vivli.org.

## Declaration of Competing Interest

Frank Jessen reports personal fees for advice from (2022–2026): Abbvie, AC immune, Biogen, Eli Lilly, Eisai, GE Healthcare, Grifols, Janssen-Cilag, Novo Nordisk, Priavoid, Roche, and Sanofi.

Grazia Dell’Agnello, Jennifer A. Zimmer, Christophe Sapin, Sascha Dichter, Erin Doty, Stéphane Epelbaum, Cynthia D. Evans, Paula M. Hauck, Rashna Khanna, Dawn A. Brooks, and John R. Sims are employees and shareholders of Eli Lilly and Company.

Federica Agosta is Associate Editor of *NeuroImage: Clinical* and the *European Journal of Neurology*; has received speaker honoraria from Biogen Idec, Bristol Myers Squibb, Eisai, Eli Lilly, GE Healthcare, Neuraxpharm, and Roche; and receives or has received research supports from the Italian Ministry of Health, the Italian Ministry of University and Research, AriSLA (Fondazione Italiana di Ricerca per la SLA), the European Research Council (ERC), the EU Joint Programme – Neurodegenerative Disease Research (JPND), and Foundation Research on Alzheimer Disease (France).

## Appendix Supplementary materials

Image, application 1
