# Supplementary material for: Efficacy and safety of donanemab in the European eligible population: TRAILBLAZER-ALZ 2 post-hoc analyses
Source: J Prev Alzheimers Dis. 2026 May 27;13(8):100605. doi: 10.1016/j.tjpad.2026.100605 (PMC13234467; doi:10.1016/j.tjpad.2026.100605)
Supplement: Supplementary file 1 [file mmc1.pdf]

Efficacy and safety of donanemab in the European eligible population: TRAILBLAZER-ALZ 2 post-hoc analyses

Supplement

Supplemental Methods

Participants in the long-term extension (LTE) of TRAILBLAZER-ALZ 2 were enrolled from the placebo-controlled period and were not required to meet additional eligibility criteria. Additional details can be found in the primary report (1).

As the LTE period did not include a placebo cohort, clinical progression according to the Clinical Dementia Rating Scale–Sum of Boxes score (which measures both cognition and function on a scale ranging from 0 to 18, with higher scores indicating greater impairment) was compared to a control cohort obtained from the Alzheimer's Disease Neuroimaging Initiative (ADNI) database (adni.loni.usc.edu). The ADNI was launched in 2003 as a public-private partnership, led by Principal Investigator Michael W. Weiner, MD. The primary goal of ADNI has been to test whether serial magnetic resonance imaging (MRI), positron emission tomography (PET), other biological markers, and clinical and neuropsychological assessment can be combined to measure the progression of mild cognitive impairment (MCI) and early Alzheimer's disease (AD). For up-to-date information, see www.adni-info.org.

The external ADNI data set generally represents an amyloid-targeting therapy–naïve AD population. Participants selected from the ADNI were apolipoprotein E ε4 non-carriers or heterozygotes with cognitive impairment who had a cerebrospinal fluid total tau-by-amyloid β42 ratio >0.28 (established criterion for amyloid pathology) (2). A full description of the methods used and the covariates included in the propensity score weighting procedure can be found in Zimmer et al. (1)

Supplemental Results

EU-indicated participants

Demographics and baseline characteristics, including the baseline iADRS score, were generally similar between the placebo and donanemab treatment arms (**Table S1**).

Efficacy

Clinical outcomes

At 76 weeks, the adjusted mean (SE) change from baseline in the iADRS score in the EU-indicated population was −13.5 (0.6) and −10.8 (0.6) in the placebo and donanemab arms, respectively (difference, 2.7 [95% CI: 1.0, 4.3]; p=0.001), representing a 20% (95% CI: 7.6, 31.7) slowing of disease progression (**Fig. S1A**). The effect was numerically greater in the low-medium tau subpopulation (difference, 3.6 [95% CI: 2.0, 5.2]; p<0.001; slowing of disease progression, 34% [95% CI: 18.9, 49.6]) (**Fig. S1B**). At 76 weeks, the adjusted mean change from baseline difference in the CDR-SB score between the donanemab and placebo arms was −0.7 (95% CI: −1.0, −0.4; p<0.001), with a 29% (95% CI: 17.4, 39.5) and 35% (95% CI: 19.1, 51.2) slowing of disease progression in the overall population and low-medium tau subpopulation, respectively (**Fig. S1C**, S**1D**). Similar results were observed with the ADAS-Cog$_{13}$ and ADCS-iADL clinical scales (**Table S2**).

A subgroup analysis evaluated iADRS and CDR-SB outcomes in donanemab-treated participants from the EU-indicated population who met treatment completion criteria and were switched to placebo at 24 or 52 weeks. At 76 weeks, the adjusted mean (SE) change from baseline in the iADRS score was −9.4 (0.84) for the completers in the donanemab arm versus −13.5 (0.6) in the placebo arm (difference, 4.1 [95% CI:

2.1, 6.1]; $p<0.001$), representing a 31% (95% CI: 15.4, 45.8) slowing of disease progression (**Fig. S2A**). The adjusted mean (SE) change from baseline in the CDR-SB score at week 76 was 1.5 (0.2) with donanemab versus 2.4 (0.1) with placebo (difference, −0.9 [95% CI: −1.2, −0.5]; $p<0.001$), representing a 37% (95% CI: 22.3, 52.0) slowing of disease progression (**Fig. S2B**).

CDR-SB domains

A positive treatment effect was observed for each of the six individual domains of the CDR-SB assessment. For the cognitive domains, the percent slowing was 30.4% ($p<0.001$) for memory, 24.4% ($p=0.003$) for orientation, and 31.0% ($p<0.001$) for judgement and problem solving. The percent slowing for functional domains was 17.4% ($p=0.020$) for community affairs, 29.6% ($p<0.001$) for home and hobbies, and 33.9% ($p=0.002$) for personal care (**Fig. S1E**).

Disease progression to the next clinical stage

The risk of disease progression to the next clinical stage of disease, as assessed by the CDR-G, was 38% lower with donanemab (N=672; 206 events) compared with placebo (N=701; 306 events) over the 76-week trial in the EU-indicated population (HR=0.623 [95% CI: 0.5, 0.8]; $p<0.001$) (**Fig. S1F**). The risk of progression to moderate dementia (CDR-G score ≥2) was significantly reduced by 48% (donanemab, N=652 [47 events]; placebo, N=682 [81 events]; HR=0.520 [95% CI: 0.3, 0.8]; $p=0.006$).

Disease stabilisation

An estimated 32% of donanemab-treated participants in the EU-indicated population was shown to be clinically stable (showed no decline on the CDR-SB from baseline) at 52 weeks compared with 22% of participants who received placebo ($p<0.001$).

Furthermore, 23% of donanemab-treated participants versus 17% of participants who received placebo had not progressed by 76 weeks (p=0.006).

Time saved

At 76 weeks, disease progression among participants treated with donanemab was delayed by 5.0 months (95% CI: 3.3, 6.8) in the EU-indicated population and 6.8 months (95% CI: 4.9, 8.6) in the EU-indicated low-medium tau subpopulation as assessed by the CDR-SB (**Fig. S2C**).

Biomarker outcomes

In the EU-indicated population, treatment with donanemab was significantly more effective to placebo in reducing amyloid plaque levels (p<0.001) (3), resulting in 32.5%, 69.5%, and 80.8% of participants achieving amyloid clearance (<24.1 CL) at weeks 24, 52, and 76, respectively. Other biomarker results in the EU-indicated population including plasma phosphorylated tau 217 are reported in the EMA Assessment Report (3).

Safety

In the EU-indicated population, treatment-emergent AEs (TEAEs) were reported by 81.3% and 88.9% of participants who received placebo and donanemab, respectively. The incidence of death with placebo and donanemab was 1.1% and 1.8%, respectively. Three deaths occurred in participants with serious ARIA, which has been previously reported (4, 5). Treatment discontinuation due to AEs was reported for 28 participants (3.8%) in the placebo arm and 91 participants (12.8%) in the donanemab arm. The most common TEAEs are shown in **Table S3**.

The incidence of ARIA-E based on magnetic resonance imaging or TEAE cluster was 1.8% (13/728) and 20.6% (146/710) in the placebo and donanemab arms, respectively. The incidence of symptomatic and serious ARIA-E in donanemab-treated participants was 5.6% (40/710) and 1.3% (9/710), respectively. No cases of serious or symptomatic ARIA-E were reported in the placebo arm (**Table S3**).

ARIA-H incidence was 12.2% (89/728) and 27.6% (196/710) in the placebo and donanemab arms, respectively, of which 0.3% (2/728) and 1.1% (8/710) were symptomatic and 0.0% (0/728) and 0.3% (2/710) were serious. The proportion of participants with isolated ARIA-H was 11.5% and 12.4% in the placebo and donanemab arms, respectively. Infusion-related reactions (IRRs) were reported for three participants (0.4%) who received placebo and 59 (8.3%) who received donanemab (**Table S3**).

TRAILBLAZER-ALZ 2: LTE period

Participants

Among the 717 *APOE* ε4 non-carrier or heterozygote participants who were randomised to receive donanemab during the placebo-controlled period (i.e. the early-start group), 465 (64.9%) received at least one infusion in the LTE period. Of these participants, 348 (74.8%) had their treatment assignment switched to placebo before the start of the LTE period, and 117 (25.2%) continued treatment with donanemab in the LTE until treatment course completion criteria were met. Of the 730 *APOE* ε4 non-carrier or heterozygote participants who were randomised to receive placebo during the placebo-controlled period, 537 (73.6%) entered the LTE and had their treatment assignment switched to donanemab (53.6% of LTE participants were in the delayed-start group) (**Fig. S3**).

Baseline characteristics of *APOE* ε4 non-carrier and heterozygote participants of the TRAILBLAZER-ALZ 2 LTE are presented in **Table S4**. A greater percentage of delayed-start participants used symptomatic medication for AD (acetylcholinesterase inhibitor and/or memantine: 68.0% vs 59.8%) at the start of the LTE than donanemab-treated participants from the EU-indicated population in the placebo-controlled period. For the EU-indicated population, mean (SD) clinical scores for the delayed-start group at the start of the LTE (iADRS: 93.7 [20.2]; CDR-SB: 5.8 [3.2]) also suggested greater cognitive impairment compared with baseline scores in the placebo-controlled period (iADRS: 104.3 [14.3]; CDR-SB: 4.0 [2.1]).

Efficacy

Clinical outcomes

Donanemab treatment continued to slow disease progression over time among early-start participants, with an adjusted mean treatment difference of −1.0 point (95% CI: −1.5, −0.4) in the CDR-SB score between donanemab and the weighted external ADNI control cohort at 154 weeks (**Fig. S4A**). Donanemab treatment also slowed disease progression among delayed-start participants, with an adjusted mean treatment difference of −0.6 points (95% CI: −1.2 to −0.0) in the CDR-SB score versus the weighted external ADNI control cohort at the end of the LTE (76 weeks after initiating donanemab) (**Fig. S4B**).

At the end of the LTE, the treatment difference from the ADNI control for early-start non-carrier and heterozygote participants who met treatment course completion criteria by 52 or by 76 weeks of the placebo-controlled period was −1.1 points (95% CI: −1.8, −0.4) (**Fig. S5A**) and −1.1 points (95% CI: −1.7, −0.5) (**Fig. S5B**), respectively.

Equivalent data for early-start participants of the overall main study who met treatment course completion criteria by 76 weeks of the placebo-controlled period was −1.2 points (95% CI: −1.8, −0.7) at the end of the LTE. Early-start participants showed a 29% reduced risk of progression to the next clinical stage of disease versus delayed-start participants (as assessed by the CDR-G score) (HR=0.71 [95% CI: 0.6, 0.9]; $p<0.001$) (**Fig. S4C**).

Biomarkers

Early-start participants achieved an adjusted mean (SE) reduction in amyloid plaque of −90.2 (1.0) CL at 76 weeks. Delayed-start participants achieved a mean (SE) reduction of −89.5 (0.9) CL at 154 weeks, 76 weeks after initiating donanemab. After initiating donanemab treatment, amyloid clearance (<24.1 CL) was achieved by a similar percentage of early- and delayed-start participants, respectively, at week 24 (35.2% [163/463] and 36.1% [175/485]), week 52 (68.0% [331/458] and 71.5% [306/428]), and week 76 (83.4% [387/464] and 80.3% [302/376]).

Among early-start participants who met treatment course completion by week 52, mean (SE) amyloid levels remained below 24.1 CL at 154 weeks (10.5 [14.3] CL) (**Fig. S5C**).

Safety

Safety data from the LTE period are shown in **Table S5**. A total of 15 deaths were reported: eight were reported for participants who were switched from donanemab to placebo at or before the start of the LTE period; one was reported for participants who continued to receive donanemab in the LTE period (donanemab-to-donanemab group); and six were reported for participants in the delayed-start group (placebo-to-

donanemab group). Of the six deaths in the delayed-start group, two were related to ARIA (one of which occurred following thrombolytic administration) as previously described (1, 5). No ARIA-related deaths occurred in the early-start group. The frequencies of ARIA-E, ARIA-H, and IRRs among early-start participants who switched to placebo were 1.1%, 13.5%, and 0.6%, respectively, with similar frequencies reported for participants who received placebo during the placebo-controlled period (1.8% for ARIA-E, 12.2% for ARIA-H, and 0.4% for IRRs). During the LTE, the frequencies of ARIA-E, ARIA-H, and IRRs among participants who continued donanemab treatment were generally lower than those of donanemab-treated participants during the placebo-controlled period (7.7%, 27.4%, and 5.1%, respectively). In the delayed-start group, TEAEs and serious AEs were reported for 86.2% and 20.1% of participants, respectively. ARIA-E, ARIA-H, and IRRs occurred in 21.8%, 35.6%, and 6.9% of participants in the delayed-start group. The safety profile among participants in the delayed-start group during the LTE was generally comparable to that among donanemab-treated participants in the initial placebo-controlled period.

Supplemental Tables and Figures

Table S1. Demographics and baseline characteristics of the EU-indicated population.

	EU-indicated population			
	Overall		Low-medium tau	
**Variable**	**Placebo (N=730)**	**Donanemab (N=717)**	**Placebo (N=494)**	**Donanemab (N=498)**
Sex, n (%)				
Female	426 (58.4)	414 (57.7)	274 (55.5)	275 (55.2)
Male	304 (41.6)	303 (42.3)	220 (44.5)	223 (44.8)
Age, mean (SD), years	73.7 (6.1)	73.5 (6.2)	75.0 (5.6)	74.9 (5.6)
Country, n (%)				
Australia	4 (0.5)	12 (1.7)	1 (0.2)	9 (1.8)
Canada	59 (8.1)	54 (7.5)	43 (8.7)	41 (8.2)
Czech Republic	10 (1.4)	6 (0.8)	8 (1.6)	3 (0.6)
Japan	38 (5.2)	37 (5.2)	32 (6.5)	32 (6.4)
Netherlands	7 (1.0)	10 (1.4)	4 (0.8)	5 (1.0)
Poland	70 (9.6)	64 (8.9)	47 (9.5)	43 (8.6)
United Kingdom	21 (2.9)	11 (1.5)	16 (3.2)	11 (2.2)
United States	521 (71.4)	523 (72.9)	343 (69.4)	354 (71.1)
Race, n (%)[a]				
Asian	42 (5.8)	49 (6.8)	34 (6.9)	40 (8.0)
Black or African American	18 (2.5)	16 (2.2)	14 (2.8)	14 (2.8)
White	669 (91.6)	649 (90.6)	446 (90.3)	443 (89.0)
American Indian or Alaska Native	0	2 (0.3)	0	1 (0.2)
Multiple	1 (0.1)	0	NA	NA
Missing	0	1 (0.1)	NA	NA
Ethnicity, n (%)[b]				
Hispanic/Latino	33 (6.4)	29 (5.6)	24 (7.0)	21 (5.9)
Not Hispanic/Latino	486 (93.6)	492 (94.4)	318 (93.0)	332 (94.1)
Education of ≥13 years, n (%)	529 (72.6)	505 (70.4)	347 (70.4)	342 (68.7)
APOE ε4 carrier, n (%)	475 (65.4)	455 (63.7)	327 (66.6)	331 (66.6)
ε2/ε2	1 (0.1)	0	1 (0.2)	0
ε2/ε3	20 (2.8)	18 (2.5)	14 (2.9)	10 (2.0)
ε2/ε4	25 (3.4)	22 (3.1)	19 (3.9)	17 (3.4)
ε3/ε3	230 (31.7)	241 (33.8)	149 (30.3)	156 (31.4)
ε3/ε4	450 (62.0)	433 (60.6)	308 (62.7)	314 (63.2)
ε4/ε4	—	—	—	—
Missing	4 (0.6)	3 (0.4)	3 (0.6)	1 (0.2)
AChEI/memantine use, n (%)	451 (61.8)	429 (59.8)	283 (57.3)	275 (55.2)

Variable	EU-indicated population Overall Placebo (N=730)	Overall Donanemab (N=717)	Low-medium tau Placebo (N=494)	Low-medium tau Donanemab (N=498)
**Clinical scales**[c]				
iADRS score, mean (SD)	103.6 (14.2)	104.3 (14.3)	105.4 (14.1)	105.7 (14.3)
CDR-SB score, mean (SD)	4.0 (2.1)	4.0 (2.1)	3.7 (2.1)	3.8 (2.2)
ADAS-Cog$_{13}$ score, mean (SD)	29.1 (9.0)	28.6 (8.9)	27.7 (8.5)	27.4 (8.7)
ADCS-ADL total score, mean (SD)	66.2 (8.6)	66.4 (8.5)	66.7 (8.8)	66.6 (8.7)
ADCS-iADL score, mean (SD)	47.7 (8.0)	47.9 (7.9)	48.2 (8.1)	48.1 (8.1)
MMSE total score, mean (SD)[d]	22.1 (3.9)	22.5 (3.8)	22.7 (3.8)	23.0 (3.7)
MMSE category, n (%)[e]				
≥27	119 (16.3)	119 (16.6)	101 (20.5)	96 (19.3)
20–26	610 (83.7)	597 (83.3)	392 (79.5)	401 (80.5)
<20	0	1 (0.1)	0	1 (0.2)
CDR-G score, n (%)				
0	3 (0.4)	2 (0.3)	3 (0.6)	2 (0.4)
0.5	439 (60.7)	422 (59.8)	317 (64.6)	317 (64.4)
1	260 (36.0)	259 (36.7)	157 (32.0)	155 (31.5)
2	21 (2.9)	23 (3.3)	14 (2.9)	18 (3.7)
**Biomarker measures, mean (SD)**				
Amyloid plaque level, Centiloids[e,f]	102.2 (34.3)	103.6 (34.5)	101.1 (34.7)	102.4 (34.9)
Tau[e,g]	1.34 (0.27)	1.34 (0.25)	1.21 (0.13)	1.21 (0.12)

Note: Numbers of participants with non-missing data were used as denominators to calculate percentages. The analysis included participants with missing *APOE* ε4 genotype data.

[a]Participants self-reported race data within fixed categories. [b]Ethnicity reporting was limited to only participants in the United States/Puerto Rico. [c]Clinical outcome ranges were as follows: ADAS-Cog$_{13}$ scores range from 0 to 85, with higher scores indicating greater overall cognition deficit; ADCS-ADL scores range from 0 to 78, with lower scores indicating greater level of impairment; ADCS-iADL scores range from 0 to 59, with lower scores indicating greater impairment in daily function; CDR-G scores range from 0 (no dementia) to 3 (severe dementia); CDR-SB scores range from 0 to 18, with higher scores indicating greater clinical impairment; iADRS scores range from 0 to 144, with lower scores indicating greater impairment; and MMSE scores range from 0 to 30, with lower scores indicating greater level of impairment. [d]Last non-missing MMSE score before or at the start of study treatment. [e]Based on screening data. [f]Assessed with 18F-florbetapir or 18F-florbetaben PET scan. [g]Assessed with 18F-flortaucipir PET scan. Global tau uptake was measured using a composite AD-signature weighted neocortical standardised uptake value ratio with white matter signal reference.

AChEI: acetylcholinesterase inhibitor; AD: Alzheimer's disease; ADAS-Cog$_{13}$: 13-item cognitive subscale of the Alzheimer's Disease Assessment Scale; ADCS-ADL: Alzheimer's Disease Cooperative Study–Activities of Daily Living; ADCS-iADL: Alzheimer's Disease Cooperative Study–Instrumental Activities of Daily Living; *APOE*: apolipoprotein E; CDR-G: Clinical Dementia Rating Scale–Global; CDR-SB: Clinical Dementia Rating Scale–Sum of Boxes; EU: European Union; iADRS: integrated Alzheimer's Disease Rating Scale; MMSE: Mini–Mental State Examination; N: number of participants in the analysis population; n: number of participants within each specific category; PET: positron emission tomography; SD: standard deviation.

Fig. S1. Clinical efficacy in the EU-indicated population.

Donanemab was significantly better than to placebo in slowing disease progression as measured with the iADRS (A–B) and CDR-SB (C–D). Compared to the EU-indicated overall population (A, C), non-carriers and heterozygotes with low-medium tau at screening (B, D) had numerically greater slowing of disease progression with donanemab compared to placebo. *p<0.05, **p<0.01, ***p<0.001, ****p<0.0001.
(E) Assessment of individual CDR-SB domains at week 76 revealed a clinically meaningful impact across all domains. (F) Cumulative hazard ratio indicating risk of progression to the next stage as assessed using the CDR-G score (range 0–3; higher scores indicate greater impairment). There were 306 events among 701 participants in the placebo arm and 206 events among 672 participants in the donanemab arm. The analysis was conducted using a Cox proportional hazards model with ARIA and deaths counted as progression as required by the Committee for Medicinal Products for Human Use.

Notes: Analyses presented in all panels except for (F) included participants with missing *APOE* ε4 genotype data and were conducted using conservative hybrid methodology for handling missing data (multiple imputation with jump-to-reference and copy increment reference methods). *APOE*: apolipoprotein E; ARIA: amyloid-related imaging abnormalities; CDR-G: Clinical Dementia Rating Scale–Global; CDR-SB: Clinical Dementia Rating Scale–Sum of Boxes; CI: confidence interval; d: days; EU: European Union; iADRS: integrated Alzheimer’s Disease Rating Scale; N: number of participants; n: number of participants at risk; SE: standard error.

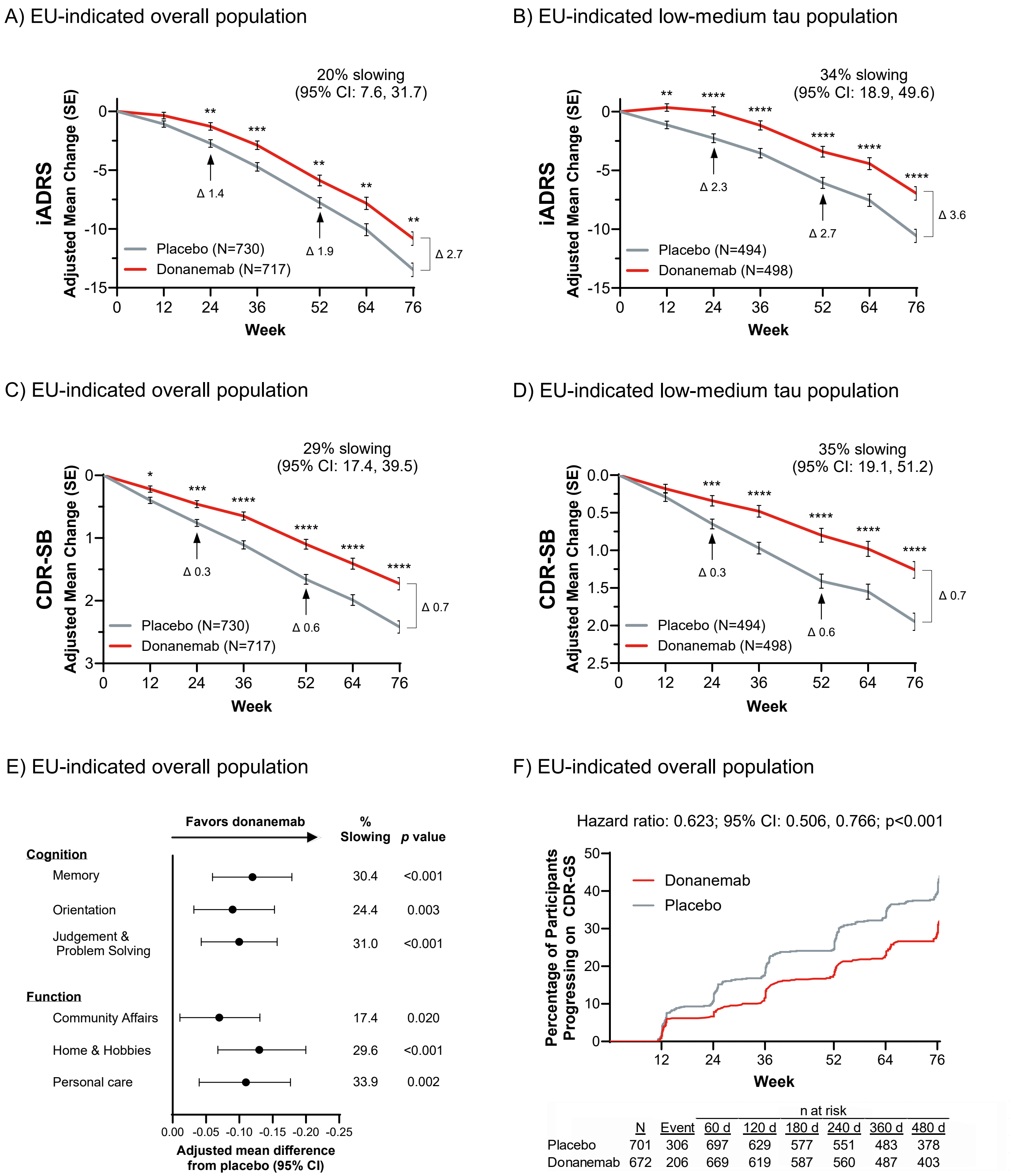
A) EU-indicated overall population
20% slowing
(95% CI: 7.6, 31.7)
iADRS
Adjusted Mean Change (SE)
Placebo (N=730)
Donanemab (N=717)
Δ 1.4
Δ 1.9
Δ 2.7
Week
B) EU-indicated low-medium tau population
34% slowing
(95% CI: 18.9, 49.6)
iADRS
Adjusted Mean Change (SE)
Placebo (N=494)
Donanemab (N=498)
Δ 2.3
Δ 2.7
Δ 3.6
Week
C) EU-indicated overall population
29% slowing
(95% CI: 17.4, 39.5)
CDR-SB
Adjusted Mean Change (SE)
Placebo (N=730)
Donanemab (N=717)
Δ 0.3
Δ 0.6
Δ 0.7
Week
D) EU-indicated low-medium tau population
35% slowing
(95% CI: 19.1, 51.2)
CDR-SB
Adjusted Mean Change (SE)
Placebo (N=494)
Donanemab (N=498)
Δ 0.3
Δ 0.6
Δ 0.7
Week
E) EU-indicated overall population
Favors donanemab
% Slowing
p value
Cognition
Memory 30.4 <0.001
Orientation 24.4 0.003
Judgement & Problem Solving 31.0 <0.001
Function
Community Affairs 17.4 0.020
Home & Hobbies 29.6 <0.001
Personal care 33.9 0.002
0.00 -0.05 -0.10 -0.15 -0.20 -0.25
Adjusted mean difference from placebo (95% CI)
F) EU-indicated overall population
Hazard ratio: 0.623; 95% CI: 0.506, 0.766; p<0.001
Percentage of Participants Progressing on CDR-GS
Donanemab
Placebo
Week
n at risk
N Event 60 d 120 d 180 d 240 d 360 d 480 d
Placebo 701 306 697 629 577 551 483 378
Donanemab 672 206 669 619 587 560 487 403

Table S2. *Impact of imputation method on clinical outcomes in the EU-indicated population.*

	Difference from placebo at 76 weeks in the EU-indicated population					
	Original statistical methodology[a]			Conservative methodology[b]		
	**Parameter (95% CI)**	***p* value**	**Percent slowing[c] (95% CI)**	**Parameter (95% CI)**	***p* value**	**Percent slowing[c] (95% CI)**
**Clinical scale**						
iADRS	3.38 (1.83, 4.92)[d]	<0.001[d]	24.9% (13.3, 36.4)[d]	2.65 (1.04, 4.26)[e]	0.001[e]	19.6% (7.6, 31.7)[e]
CDR-SB	−0.77 (−1.04, −0.49)[e]	<0.001[e]	31.5% (19.8, 43.1)[e]	−0.69 (−0.95, −0.43)[e]	<0.001[e]	28.5% (17.4, 39.5)[e]
ADAS-Cog$_{13}$	−1.69 (−2.52, −0.86)[d]	<0.001[d]	24.0% (12.1, 35.9)[d]	−1.35 (−2.19, −0.51)[e]	0.002[e]	19.3% (7.2, 31.3)[e]
ADCS-iADL	1.76 (0.81, 2.72)[d]	<0.001[d]	27.9% (12.5, 43.4)[d]	1.46 (0.50, 2.42)[e]	0.003[e]	23.0% (7.7, 38.2)[e]
MMSE	0.55 (0.15, 0.95)[d]	0.007[d]	18.4% (4.9, 32.0)[d]	0.40 (−0.02, 0.81)[e]	0.059[e]	13.2% (−0.5, 26.9)[e]
Hazard ratio of progression CDR-G score[f]	0.597 (0.474, 0.751)[g]	<0.001	-	0.623 (0.506, 0.766)[h]	<0.001	-
Delayed disease progression (CDR-SB)[i]	-	-	-	-		-
Time saved after 76 weeks (in months)	5.82 (4.20, 7.44)	<0.001	-	5.02 (3.28, 6.77)	<0.001	-
Percent time savings	33.17 (23.95, 42.4)		-	28.75 (18.77, 38.74)		-

Notes: For clinical scales, the parameter is 76-week adjusted mean CFB difference from placebo. For hazard ratio of progression, the parameter is the hazard ratio through 76 weeks. For delayed disease progression, the parameter for time is months and the parameter for percent time savings is percent. Participants with missing *APOE* ε4 genotype data were included in all analyses except for hazard ratio of progression using conservative methodology. . ADAS-Cog$_{13}$ scores range from 0 to 85, with higher scores indicating greater overall cognition deficit; ADCS-iADL scores range from 0 to 59, with lower scores indicating greater impairment in daily function; CDR-G scores range from 0 to 3 as follows: 0 = no impairment, 0.5 = mild cognitive impairment, 1 = mild dementia, 2 = moderate dementia, and 3 = severe dementia; ≥2 means progression to moderate or severe dementia; CDR-SB scores range from 0 to 18, with higher scores indicating greater clinical impairment; iADRS scores range from 0 to 144, with lower scores indicating greater impairment; and MMSE scores range from 0 to 30, with lower scores indicating greater level of impairment.

[a]No formal imputation was used. [b]Conservative hybrid imputation was used for clinical scales and delayed disease progression analyses (if the participant discontinued due to serious/severe/symptomatic ARIA-E/ARIA-H or death, the jump-to-reference method was used; the copy increment reference method was used for all other missing data). For hazard ratio of progression, conservative methodology means that discontinuations due to death and ARIA are classified as events.[c]Percent slowing was calculated by dividing the adjusted mean CFB treatment differences at 76 weeks by the adjusted mean CFB with placebo at 76 weeks and multiplying by 100. The 95% CIs were estimated using the Delta method. [d]NCS2: Adjusted mean change from baseline, SE, 95% CI, and *p* values are derived using the NCS model with 2 degrees of freedom. The model was adjusted for basis expansion terms (two terms), basis expansion term-by-treatment interaction and covariates for age at baseline, pooled investigator, baseline tau category, and baseline AChEI/memantine use. [e]MMRM: Adjusted mean CFB, 95% CIs, and *p* values were derived using an MMRM with treatment, visit, baseline tau category, pooled investigator, and concomitant use of symptomatic treatment (acetylcholinesterase inhibitors and/or memantine) as factors, baseline age and baseline score as covariates, and treatment-by-visit and baseline score-by-visit interaction terms. The 95% CIs for adjusted mean changes were calculated with the normal approximation method. [f]Hazard ratio, 95% CI, and *p* value were calculated using a Cox proportional hazards model stratified by pooled investigator and baseline tau level that included baseline covariates of age,

AChEI/memantine use, and clinical outcome score. Events/number of participants for placebo and donanemab arms were [g]241/701 and 153/672; [h]306/701 and 206/672. [i]Calculated using the progression model for repeated measures.

AChEI: acetylcholinesterase inhibitor; ADAS-Cog$_{13}$: 13-item cognitive subscale of the Alzheimer's Disease Assessment Scale; ADCS-iADL: Alzheimer's Disease Cooperative Study–Instrumental Activities of Daily Living; *APOE*: apolipoprotein E; ARIA-E: amyloid-related imaging abnormality–edema/effusion; ARIA-H: amyloid-related imaging abnormality–microhemorrhages and hemosiderin deposits; CDR-G: Clinical Dementia Rating Scale–Global; CDR-SB: Clinical Dementia Rating Scale–Sum of Boxes; CFB: change from baseline; CI: confidence interval; EU: European Union; iADRS: integrated Alzheimer's Disease Rating Scale; MMRM: mixed model for repeated measures; MMSE: Mini–Mental State Examination; NCS: natural cubic spline.

Fig. S2. Additional clinical outcomes in the EU-indicated population.

Time course of (A) iADRS and (B) CDR-SB scores of donanemab-treated participants who met treatment completion criteria and switched to placebo at 24 or 52 weeks compared to the entire placebo arm. The analysis was conducted using conservative methods for handling missing data (multiple imputation with jump-to-reference and copy increment reference methods). All analyses included participants with missing *APOE* ε4 genotype data. **p*<0.05, ***p*<0.01, ****p*<0.001, *****p*<0.0001. (C) Results of the CDR-SB progression model for repeated measures analysis with hybrid imputation illustrating the time saved with donanemab treatment. *APOE*: apolipoprotein E; CDR-SB: Clinical Dementia Rating Scale–Sum of Boxes; CI: confidence interval; EU: European Union; iADRS: integrated Alzheimer's Disease Rating Scale; SE: standard error.

A)

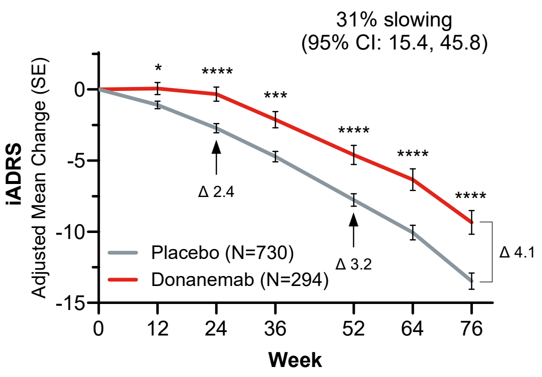

31% slowing
(95% CI: 15.4, 45.8)
iADRS
Adjusted Mean Change (SE)
0
-5
-10
-15
*
****
***
****
****
****
Δ 2.4
Δ 3.2
Δ 4.1
Placebo (N=730)
Donanemab (N=294)
0
12
24
36
52
64
76
Week

B)

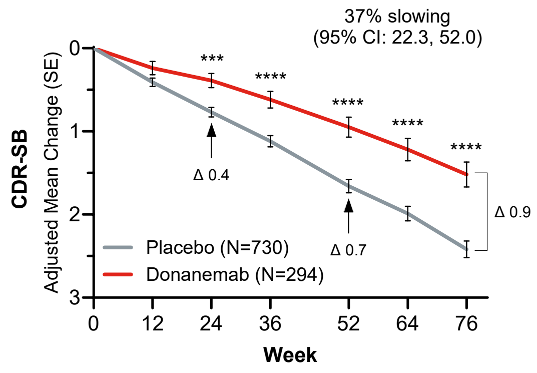

37% slowing
(95% CI: 22.3, 52.0)
CDR-SB
Adjusted Mean Change (SE)
0
1
2
3
***
****
****
****
****
Δ 0.4
Δ 0.7
Δ 0.9
Placebo (N=730)
Donanemab (N=294)
0
12
24
36
52
64
76
Week

C)

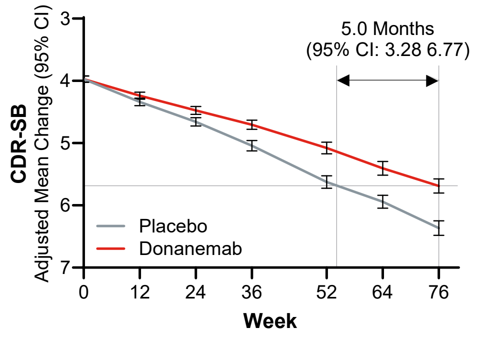

5.0 Months
(95% CI: 3.28 6.77)
CDR-SB
Adjusted Mean Change (95% CI)
3
4
5
6
7
Placebo
Donanemab
0
12
24
36
52
64
76
Week

Table S3. *Safety summary for the EU-indicated population in the placebo-controlled period.*

	EU-indicated population	
**Category,[a] n (%)**	**Placebo (N=728)**	**Donanemab (N=710)**
**Overview**		
Deaths[b]	8 (1.1)	13 (1.8)
Deaths associated with any ARIA/ICH >1 cm	0	3 (0.4)
Participants with ≥1 SAE	111 (15.2)	127 (17.9)
Treatment discontinuations due to AEs	28 (3.8)	91 (12.8)
Study discontinuations due to AEs	26 (3.6)	54 (7.6)
Participants with ≥1 TEAE	592 (81.3)	631 (88.9)
**TEAEs with ≥5% incidence in any arm[c]**		
ARIA-E	12 (1.6)	146 (20.6)
ARIA-H	50 (6.9)	121 (17.0)
COVID-19	129 (17.7)	114 (16.1)
Fall	91 (12.5)	106 (14.9)
Headache	73 (10.0)	104 (14.6)
Infusion-related reaction	3 (0.4)	59 (8.3)
Arthralgia	40 (5.5)	46 (6.5)
Diarrhoea	40 (5.5)	40 (5.6)
Dizziness	39 (5.4)	40 (5.6)
Superficial siderosis of central nervous system	6 (0.8)	40 (5.6)
Urinary tract infection	49 (6.7)	40 (5.6)
Fatigue	39 (5.4)	34 (4.8)
**ARIA overview**		
Any ARIA (ARIA-E or ARIA-H)[d]	98 (13.5)	234 (33.0)
SAE of any ARIA[e]	0	10 (1.4)
ARIA-E[d]	13 (1.8)	146 (20.6)
SAE of ARIA-E[e]	0	9 (1.3)
Asymptomatic	13 (1.8)	106 (14.9)
Symptomatic	0	40 (5.6)
Resolution of ARIA-E symptoms	—	32 (80.0)
Maximum radiographic severity[f]		
Mild	10 (1.4)	44 (6.2)
Moderate	2 (0.3)	90 (12.7)
Severe	0	10 (1.4)
ARIA-H[d]	89 (12.2)	196 (27.6)
SAE of ARIA-H[e]	0	2 (0.3)
Asymptomatic	87 (12.0)	188 (26.5)
Symptomatic	2 (0.3)	8 (1.1)
Maximum radiographic severity[f]		
Mild	70 (9.6)	102 (14.4)
Moderate	12 (1.6)	39 (5.5)
Severe	3 (0.4)	54 (7.6)
Isolated ARIA-H[d,g]	84 (11.5)	88 (12.4)
Microhemorrhage[f]	77 (10.6)	161 (22.7)
Superficial siderosis[f]	15 (2.1)	94 (13.2)
Macrohemorrhage[d]	2 (0.3)	3 (0.4)
SAE of macrohaemorrhage[e]	1 (0.1)	1 (0.1)

Note: All assessments included participants with missing *APOE* ε4 genotype data. [a]Participants may have been counted in more than one category. [b]Deaths are also included as SAEs and discontinuations due to AEs. [c]TEAEs were coded using the Medical Dictionary for Adverse Events version 25.1. [d]Based on MRI or TEAE cluster. [e]Based

on TEAE cluster. [f]Based on MRI only. [g]Isolated ARIA-H means no ARIA-E events based on MRI or TEAE cluster in the same analysis period.

AE: adverse event; *APOE*: apolipoprotein E; ARIA: amyloid-related imaging abnormalities; ARIA-E: amyloid-related imaging abnormality–edema/effusions; ARIA-H: amyloid-related imaging abnormality–microhemorrhages and hemosiderin deposits; COVID-19: coronavirus disease 2019; EU: European Union; ICH: intracerebral hemorrhage; MRI: magnetic resonance imaging; N: number of participants in the analysis population; n: number of participants within each specific category; SAE: serious adverse event; TEAE: treatment-emergent adverse event.

Figure S3 Study design

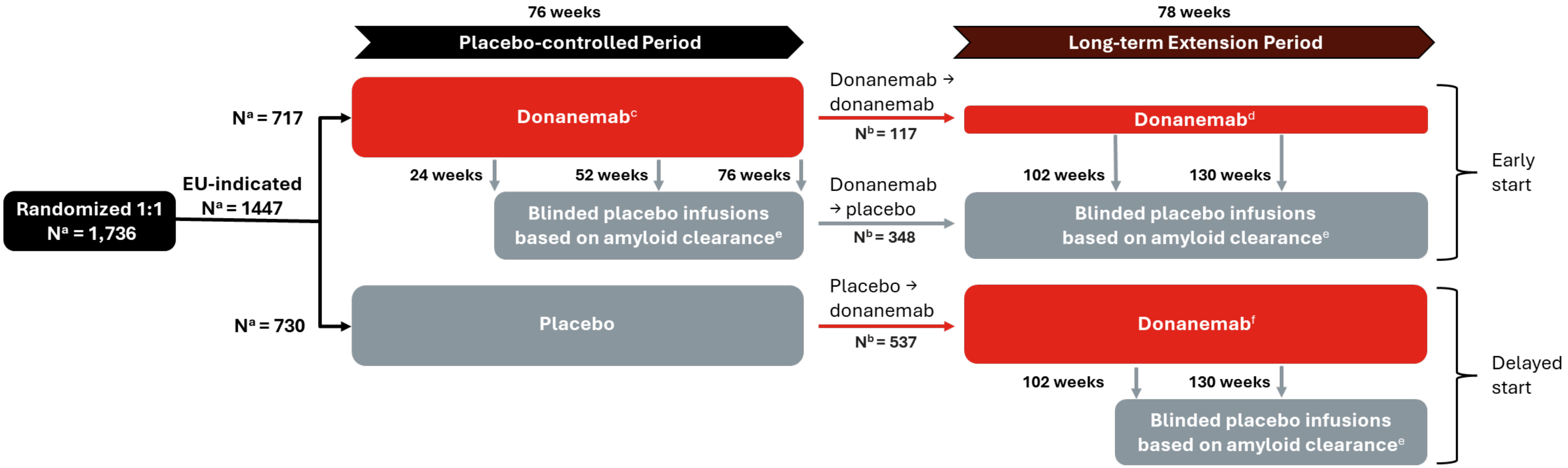

[a]Number of participants includes those with missing *APOE* ε4 genotype data [b]Number of participants (not include those with missing *APOE* ε4 genotype data) receiving at least one infusion in the LTE period; [c]700 mg Q4W for the first 3 doses and 1400 mg Q4W thereafter administered intravenously; [d]Participants randomized to donanemab during the placebo-controlled period who did not meet the treatment completion criteria by 76 weeks continued receiving donanemab Q4W; [e]Participants who met prespecified treatment completion criteria based on amyloid PET were switched in a blinded fashion to placebo Q4W (saline infusion); [f]Participants randomized to placebo Q4W during the placebo-controlled period were assigned to receive donanemab Q4W starting at Visit 22 (78 weeks after randomization) and followed the same dose titration as participants during the placebo-controlled period.

LTE: long-term extension; MRI: magnetic resonance imaging; N: number of participants; PET: positron emission tomography; Q4W: every four weeks.

Table S4. *Demographics and baseline characteristics in APOE ε4 non-carriers and heterozygotes of the LTE population*

Variable	Early-start participants dosed in the LTE		Delayed-start participants dosed in the LTE
	**Donanemab → placebo (N=348)**	**Donanemab → donanemab (N=117)**	**Placebo → donanemab (N=537)**
Sex, n (%)			
Female	196 (56.3)	63 (53.8)	311 (57.9)
Male	152 (43.7)	54 (46.2)	226 (42.1)
Age, mean (SD), years	76.7 (6.0)	72.4 (5.8)	75.6 (6.1)
Country			
Australia	8 (2.3)	1 (0.9)	3 (0.6)
Canada	23 (6.6)	10 (8.5)	40 (7.4)
Czech Republic	4 (1.1)	1 (0.9)	5 (0.9)
Japan	23 (6.6)	5 (4.3)	31 (5.8)
Netherlands	4 (1.1)	1 (0.9)	7 (1.3)
Poland	32 (9.2)	12 (10.3)	57 (10.6)
United Kingdom	6 (1.7)	1 (0.9)	16 (3.0)
United States	248 (71.3)	86 (73.5)	378 (70.4)
Race, n (%)[a]			
Asian	29 (8.4)	5 (4.3)	34 (6.3)
Black or African American	5 (1.4)	3 (2.6)	10 (1.9)
White	312 (89.9)	109 (93.2)	492 (91.6)
American Indian or Alaska Native	1 (0.3)	0	0
Multiple	0	0	1 (0.2)
Missing	1 (0.3)	0	0
Ethnicity, n (%)[b]			
Hispanic/Latino	11 (4.4)	7 (8.1)	18 (4.8)
Not Hispanic/Latino	237 (95.6)	79 (91.9)	360 (95.2)
Education of ≥13 years, n (%)	238 (68.4)	87 (74.4)	400 (74.5)
APOE ε4 carrier, n (%)	204 (58.6)	86 (73.5)	352 (65.5)
ε2/ε2	0	0	1 (0.2)
ε2/ε3	11 (3.2)	0	15 (2.8)
ε2/ε4	11 (3.2)	4 (3.4)	18 (3.4)
ε3/ε3	133 (38.2)	31 (26.5)	169 (31.5)
ε3/ε4	193 (55.5)	82 (70.1)	334 (62.2)
ε4/ε4	—	—	—
AChEI and/or memantine use, n (%)	216 (62.1)	76 (65.0)	365 (68.0)
**Clinical scales[c]**			
iADRS score, mean (SD)	97.8 (19.8)	99.6 (20.4)	93.7 (20.2)

Variable	Early-start participants dosed in the LTE		Delayed-start participants dosed in the LTE
	**Donanemab → placebo (N=348)**	**Donanemab → donanemab (N=117)**	**Placebo → donanemab (N=537)**
CDR-SB score, mean (SD)	5.0 (3.0)	4.9 (3.0)	5.8 (3.2)
ADAS-Cog13 score, mean (SD)	32.1 (12.0)	31.5 (11.3)	34.6 (11.9)
ADCS-ADL total score, mean (SD)	629 (11.6)	64.2 (12.7)	61.2 (12.0)
ADCS-iADL score, mean (SD)	44.8 (10.4)	46.0 (11.1)	43.2 (10.6)
MMSE total score, mean (SD)[d]	21.0 (5.3)	21.1 (5.1)	19.7 (5.59)
MMSE category, n (%)[d]			
≥27	64 (18.4)	15 (12.8)	60 (11.2)
20–26	151 (43.4)	57 (48.7)	232 (43.2)
<20	133 (38.2)	45 (38.5)	245 (45.6)
CDR-G score, n (%)			
0	6 (1.7)	2 (1.7)	3 (0.6)
0.5	153 (44.0)	57 (48.7)	194 (36.1)
1	149 (42.8)	45 (38.5)	254 (47.3)
2	38 (10.9)	12 (10.3)	79 (14.7)
3	2 (0.6)	1 (0.9)	7 (1.3)
**Biomarker measures, mean (SD)**			
Amyloid plaque level, Centiloids[e,f]	95.9 (31.5)	123.9 (35.3)	102.7 (34.2)
Tau[e,g]	1.31 (0.24)	1.39 (0.27)	1.35 (0.26)

Note: Numbers of participants with non-missing data were used as denominators to calculate percentages. Demographics and disease characteristics are at the start of the LTE except where noted. The analysis did not include participants with missing *APOE* ε4 genotype data.

[a]Participants self-reported race data within fixed categories. [b]Ethnicity reporting was limited to only participants in the United States/Puerto Rico. [c]Clinical outcome ranges were as follows: ADAS-Cog13 scores range from 0 to 85, with higher scores indicating greater overall cognition deficit; ADCS-ADL scores range from 0 to 78, with lower scores indicating greater level of impairment; ADCS-iADL scores range from 0 to 59, with lower scores indicating greater impairment in daily function; CDR-G scores range from 0 (no dementia) to 3 (severe dementia); CDR-SB scores range from 0 to 18, with higher scores indicating greater clinical impairment; iADRS scores range from 0 to 144, with lower scores indicating greater impairment; and MMSE scores range from 0 to 30, with lower scores indicating greater level of impairment. [d]Last non-missing MMSE score before or at the start of study treatment in the LTE. [e]Based on screening data. [f]Assessed with 18F-florbetapir or 18F-florbetaben PET scan. [g]Assessed with 18F-flortaucipir PET scan. Global tau uptake was measured using a composite AD-signature weighted neocortical standardised uptake value ratio with white matter signal reference.

AChEI: acetylcholinesterase inhibitor; AD: Alzheimer's disease; ADAS-Cog13: 13-item cognitive subscale of the Alzheimer's Disease Assessment Scale; ADCS-ADL: Alzheimer's Disease Cooperative Study–Activities of Daily Living; ADCS-iADL: Alzheimer's Disease Cooperative Study–Instrumental Activities of Daily Living; *APOE*: apolipoprotein E; CDR-G: Clinical Dementia Rating Scale–Global; CDR-SB: Clinical Dementia Rating Scale–Sum of Boxes; iADRS: integrated Alzheimer's Disease Rating Scale; LTE: long-term extension; MMSE: Mini–Mental State Examination; N: number of participants in the analysis population; n: number of participants in the specified category; PET: positron emission tomography; SD: standard deviation.

Fig. S4. Clinical efficacy in APOE ε4 non-carriers and heterozygotes in the long-term extension.

Clinical efficacy was measured by the mean change from baseline in the CDR-SB scores for participants in the (A) early-start and (B) delayed-start groups versus the external ADNI control cohort. The analyses for panel (A) and (B) did not include participants with missing *APOE* ε4 genotype data. (C) Disease progression was measured using CDR-G scores among early-start versus delayed-start groups. Early-start participants showed a 29% reduced risk of progression to the next clinical stage of the disease versus delayed-start participants. The disease progression analysis included participants with missing *APOE* ε4 genotype data. [a]Hazard ratio, 95% CI, and *p* value were calculated using a Cox proportional hazards model. The model was stratified by pooled investigator and baseline tau level and included baseline covariates of age, CDR-G score, and acetylcholinesterase inhibitor/memantine use.

ADNI: Alzheimer's Disease Neuroimaging Initiative; *APOE*: apolipoprotein E; CDR-G: Clinical Dementia Rating Scale–Global; CDR-SB: Clinical Dementia Rating Scale–Sum of Boxes; CI: confidence interval; d: days; ESS: effective sample size; N: number of participants; n: number of participants at risk; SE: standard error.

A)

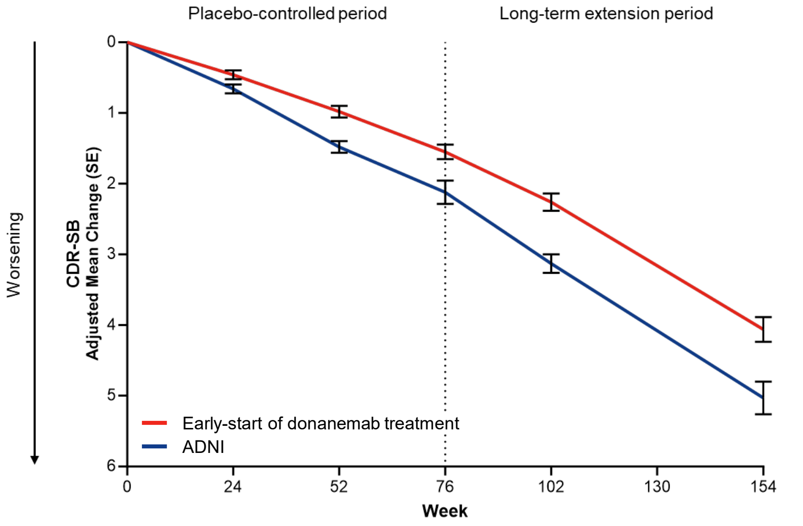

	0	24	52	76	102	154
Early-start vs ADNI difference (95% CI)		-0.2 (-0.4, -0.1)	-0.5 (-0.7, -0.3)	-0.6 (-0.9, -0.2)	-0.9 (-1.2, -0.5)	-1.0 (-1.5, -0.4)
Early-start (N)	661	610	547	506	428	352
ADNI (ESS)	210	198	183	153	141	95

B)

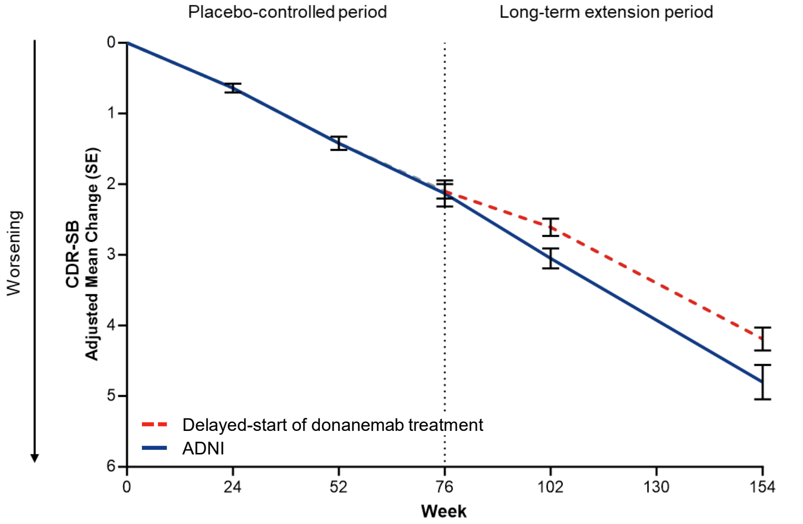

	0	24	52	76	102	154
Delayed-start vs ADNI difference (95% CI)		0 (-0.2, 0.2)	-0.0 (-0.2, 0.2)	-0.0 (-0.4, 0.4)	-0.4 (-0.8, -0.1)	-0.6 (-1.2, -0.0)
Delayed-start (N)	535	518	515	527	476	381
ADNI (ESS)	234	220	204	172	159	109

C)

Hazard ratio: 0.71 (95% CI, 0.592, 0.852); p <0.001[a]

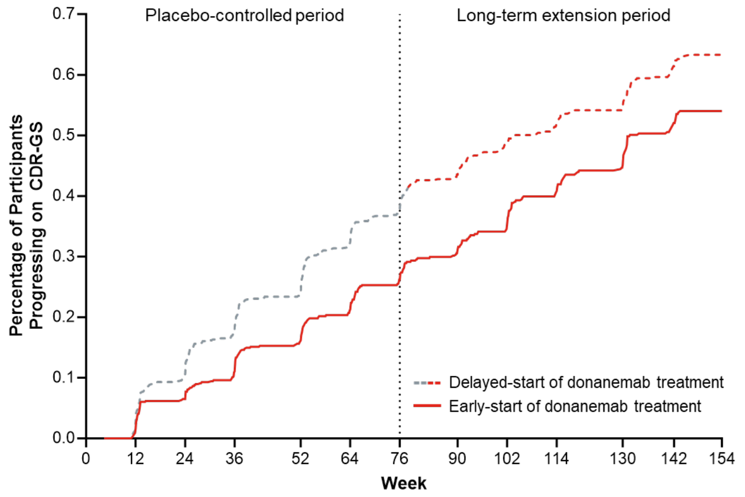

			n at risk											
	N	Event	84 d	168 d	252 d	364 d	448 d	532 d	630 d	715 d	798 d	910 d	995 d	1078 d
Delayed-start	711	378	683	611	539	480	405	363	306	271	246	216	171	132
Early-start	690	285	654	608	547	482	432	387	328	296	266	235	197	161

Fig. S5. Efficacy among early-start participants who met treatment completion criteria by the start of the LTE.

CDR-SB trajectories of *APOE* ε4 non-carrier and heterozygote participants of the TRAILBLAZER-ALZ 2 long-term extension early-start group who met treatment course completion criteria based on amyloid PET scans by (A) 52 weeks (based on amyloid PET at weeks 24 or 52) of the placebo-controlled period and (B) 76 weeks (based on amyloid PET at weeks 24, 52, or 76) were compared to the external ADNI control. Propensity score weights were estimated for the ADNI cohort with the average treatment effect among the treated estimand using the inverse probability weighting method from the generalised linear model. Change from baseline in the CDR-SB score was estimated using a mixed model for repeated measures using ADNI weights. The CDR-SB analyses did not include participants with missing *APOE* ε4 genotype data. (C) Amyloid plaque trajectory of early-start participants who completed the treatment course by 52 weeks of the placebo-controlled period showing slow reaccumulation through the long-term extension. This analysis included participants with missing *APOE* ε4 genotype data.

ADNI: Alzheimer's Disease Neuroimaging Initiative; *APOE*: apolipoprotein E; CDR-SB: Clinical Dementia Rating Scale–Sum of Boxes; CI: confidence interval; ESS: effective sample size; PET: positron emission tomography; SD: standard deviation; SE: standard error.

A)

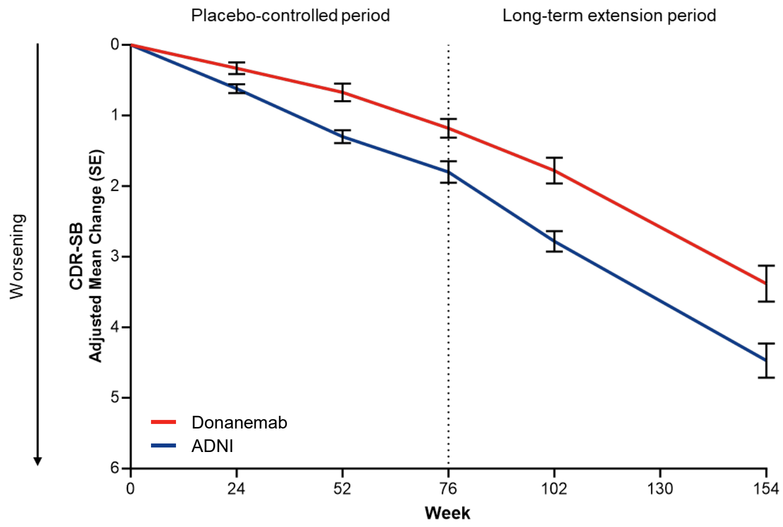

	0	24	52	76	102	130	154
Donanemab vs ADNI difference (95% CI)		-0.3 (-0.5, -0.1)	-0.6 (-0.9, -0.4)	-0.6 (-1.0, -0.2)	-1.0 (-1.4, -0.6)		-1.1 (-1.8, -0.4)
Donanemab (N)	291	285	272	259	225		178
ADNI (ESS)	210	198	183	153	141		95

B)

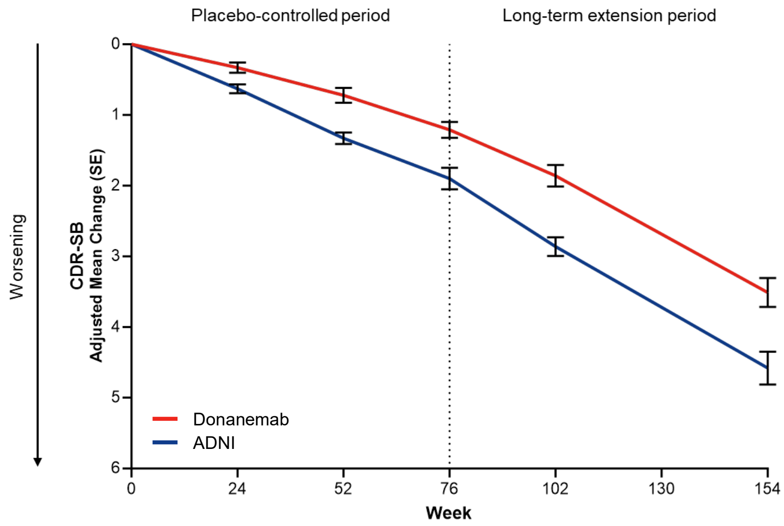

	0	24	52	76	102	130	154
Donanemab vs ADNI difference (95% CI)		-0.3 (-0.5, -0.1)	-0.6 (-0.9, -0.4)	-0.7 (-1.1, -0.3)	-1.0 (-1.4, -0.6)		-1.1 (-1.7, -0.5)
Donanemab (N)	401	390	379	366	315		253
ADNI (ESS)	210	198	183	153	141		95

C)

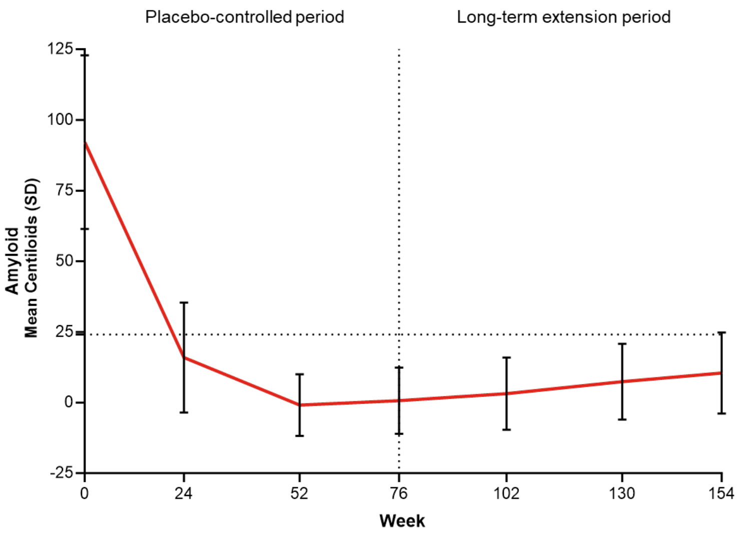

N	294	293	280	265	230	203	173

Table S5. *Safety summary for APOE ε4 non-carriers and heterozygotes in the LTE period.*

	Early-start participants dosed in the LTE[b]		Delayed-start participants dosed in the LTE[b]
**Category,[a] n (%)**	**Donanemab → placebo (N=348)**	**Donanemab → donanemab (N=117)**	**Placebo → donanemab (N=537)**
**Overview**			
Deaths[c]	8 (2.3)	1 (0.9)	6 (1.1)
Deaths associated with any ARIA/ICH >1 cm	0	0	2 (0.4)
Participants with ≥1 SAE	72 (20.7)	12 (10.3)	108 (20.1)
Treatment discontinuations due to AEs	15 (4.3)	4 (3.4)	61 (11.4)
Study discontinuations due to AEs	15 (4.3)	4 (3.4)	27 (5.0)
Participants with ≥1 TEAE[d]	281 (80.7)	96 (82.1)	463 (86.2)
**TEAEs with ≥5% incidence in any arm[d,e]**			
ARIA-H	23 (6.6)	19 (16.2)	112 (20.9)
ARIA-E	4 (1.1)	9 (7.7)	117 (21.8)
Arthralgia	13 (3.7)	8 (6.8)	28 (5.2)
Back pain	15 (4.3)	6 (5.1)	23 (4.3)
COVID-19	43 (12.4)	11 (9.4)	75 (14.0)
Dizziness	24 (6.9)	4 (3.4)	29 (5.4)
Fall	47 (13.5)	14 (12.0)	100 (18.6)
Headache	15 (4.3)	10 (8.5)	50 (9.3)
Fatigue	4 (1.1)	6 (5.1)	15 (2.8)
Infusion-related reaction	2 (0.6)	6 (5.1)	37 (6.9)
Nasopharyngitis	13 (3.7)	4 (3.4)	29 (5.4)
Superficial siderosis of central nervous system	4 (1.1)	2 (1.7)	28 (5.2)
Upper respiratory tract infection	15 (4.3)	7 (6.0)	29 (5.4)
Urinary tract infection	29 (8.3)	10 (8.5)	45 (8.4)
**ARIA overview**			
Any ARIA (ARIA-E or ARIA-H)[f]	50 (14.4)	36 (30.8)	211 (39.3)
SAE of any ARIA[g]	0	0	7 (1.3)
ARIA-E[f]	4 (1.1)	9 (7.7)	117 (21.8)
Symptomatic	1 (0.3)	1 (0.9)	29 (5.4)
SAE of ARIA-E[g]	0	0	7 (1.3)
ARIA-H[f]	47 (13.5)	32 (27.4)	191 (35.6)
Symptomatic	1 (0.3)	0	2 (0.4)
SAE of ARIA-H[g]	0	0	0
Macrohemorrhage[f]	0	0	4 (0.7)
SAE of macrohemorrhage[g]	0	0	1 (0.2)

Note: All assessments included participants with missing *APOE* ε4 genotype data. [a]Participants may have been counted in more than one category. [b]Includes participants who received at least one infusion during the LTE. [c]Deaths are also included as SAEs and discontinuations due to AEs. [d]TEAEs are baseline AEs defined as all ongoing AEs at the first LTE dose; the postbaseline period started the day of the first LTE infusion and ended at the earlier date of study withdrawal or completion, the end of the LTE period + 57 days, or at data cutoff. [e]TEAEs were coded using the Medical Dictionary for Adverse Events version 27.1. [f]Based on magnetic resonance imaging or TEAE cluster. [g]Based on TEAE cluster.

AE: adverse event; *APOE*: apolipoprotein E; ARIA: amyloid-related imaging abnormalities; ARIA-E: amyloid-related imaging abnormality–edema/effusion; ARIA-H: amyloid-related imaging abnormality–microhemorrhages and hemosiderin deposits; COVID-19: coronavirus disease 2019; ICH: intracerebral hemorrhage; LTE: long-term extension; N: number of participants in the analysis population; n: number of participants within each specific category; SAE: serious adverse event; TEAE: treatment-emergent adverse event.

References

1. Zimmer JA, Sims JR, Evans CD, et al. Donanemab in early symptomatic Alzheimer's disease: results from the TRAILBLAZER-ALZ 2 long-term extension. J Prev Alzheimers Dis 2025:100446. doi:10.1016/j.tjpad.2025.100446

2. Roche Diagnostics. Elecsys Total-Tau CSF. Method sheet. 2023. Accessed September 19, 2025. https://assets.roche.com/f/173850/x/9ad8e46bb4/total-tau-08846685190-en-can.pdf

3. European Medicines Agency. Kisunla - Assessment report 2025. Accessed January 12, 2026. https://www.ema.europa.eu/en/documents/assessment-report/kisunla-epar-public-assessment-report_en.pdf

4. Sims JR, Zimmer JA, Evans CD, et al. Donanemab in Early Symptomatic Alzheimer Disease: The TRAILBLAZER-ALZ 2 Randomized Clinical Trial. JAMA 2023;330:512-527. doi:10.1001/jama.2023.13239

5. Zimmer JA, Ardayfio P, Wang H, et al. Amyloid-related imaging abnormalities with donanemab in early symptomatic Alzheimer disease: secondary analysis of the TRAILBLAZER-ALZ and ALZ 2 randomized clinical trials. JAMA Neurol 2025;82:461–469. doi:10.1001/jamaneurol.2025.0065
